# Automatic patient-level recognition of four *Plasmodium* species on thin blood smear by a real-time detection transformer (RT-DETR) object detection algorithm: a proof-of-concept and evaluation

**DOI:** 10.1128/spectrum.01440-23

**Published:** 2024-01-03

**Authors:** Emilie Guemas, Baptiste Routier, Théo Ghelfenstein-Ferreira, Camille Cordier, Sophie Hartuis, Bénédicte Marion, Sébastien Bertout, Emmanuelle Varlet-Marie, Damien Costa, Grégoire Pasquier

**Affiliations:** 1 Department of Parasitology and Mycology, Academic Hospital (CHU) of Toulouse, Toulouse, France; 2 Toulouse Institute for Infectious and Inflammatory Diseases (Infinity), CNRS UMR5051, INSERM UMR1291, UPS, Toulouse, France; 3 Laboratory of Parasitology-Mycology, EA7510 ESCAPE, University Hospital of Rouen, University of Rouen Normandie, Normandie, France; 4 Université de Paris Cité, Laboratoire de Parasitologie-Mycologie, Groupe Hospitalier Saint-Louis-Lariboisière-Fernand-Widal, Assistance Publique-Hôpitaux de Paris (AP-HP), Paris, France; 5 Laboratory of Parasitology-Mycology, INSERM U1285, Unité de Glycobiologie Structurale et Fonctionnelle (CNRS UMR 8576), University Hospital (CHU) of Lille, University of Lille, Lille, France; 6 Nantes University,Academic Hospital (CHU) of Nantes,Cibles et Médicaments des Infections et de l'Immunité, IICiMed, UR1155, Nantes, France; 7 Department of Physical Chemistry and Biophysics, Academic Hospital (CHU) of Montpellier, University of Montpellier, National Reference Centre (CNR) for Paludism, Montpellier, France; 8 Department of Parasitology/Mycology, Academic Hospital (CHU) of Montpellier, University of Montpellier, National Reference Centre (CNR) for Paludism, Montpellier, France; 9 Laboratory of Parasitology/Mycology, UMI 233 TransVIHMI, University of Montpellier, IRD, INSERM U1175, Montpellier, France; Quest Diagnostics Nichols Institute, Chantilly, Virginia, USA

**Keywords:** *Plasmodium*, artificial intelligence, deep learning, machine learning, object detection, YOLO, RT-DETR, diagnosis, thin blood smear

## Abstract

Malaria remains a global health problem, with 247 million cases and 619,000 deaths in 2021. Diagnosis of *Plasmodium* species is important for administering the appropriate treatment. The gold-standard diagnosis for accurate species identification remains the thin blood smear. Nevertheless, this method is time-consuming and requires highly skilled and trained microscopists. To overcome these issues, new diagnostic tools based on deep learning are emerging. This study aimed to evaluate the performances of a real-time detection transformer (RT-DETR) object detection algorithm to discriminate *Plasmodium* species on thin blood smear images. The algorithm was trained and validated on a data set consisting in 24,720 images from 475 thin blood smears corresponding to 2,002,597 labels. Performances were calculated with a test data set of 4,508 images from 170 smears corresponding to 358,825 labels coming from six French university hospitals. At the patient level, the RT-DETR algorithm exhibited an overall accuracy of 79.4% (135/170) with a recall of 74% (40/54) and 81.9% (95/116) for negative and positive smears, respectively. Among *Plasmodium-*positive smears, the global accuracy was 82.7% (91/110) with a recall of 90% (38/42), 81.8% (18/22), and 76.1% (35/46) for *P. falciparum*, *P. malariae,* and *P. ovale/vivax,* respectively. The RT-DETR model achieved a World Health Organization (WHO) competence level 2 for species identification. Besides, the RT-DETR algorithm may be run in real-time on low-cost devices such as a smartphone and could be suitable for deployment in low-resource setting areas lacking microscopy experts.

**IMPORTANCE:**

Malaria remains a global health problem, with 247 million cases and 619,000 deaths in 2021. Diagnosis of *Plasmodium* species is important for administering the appropriate treatment. The gold-standard diagnosis for accurate species identification remains the thin blood smear. Nevertheless, this method is time-consuming and requires highly skilled and trained microscopists. To overcome these issues, new diagnostic tools based on deep learning are emerging. This study aimed to evaluate the performances of a real-time detection transformer (RT-DETR) object detection algorithm to discriminate *Plasmodium* species on thin blood smear images. Performances were calculated with a test data set of 4,508 images from 170 smears coming from six French university hospitals. The RT-DETR model achieved a World Health Organization (WHO) competence level 2 for species identification. Besides, the RT-DETR algorithm may be run in real-time on low-cost devices and could be suitable for deployment in low-resource setting areas.

## INTRODUCTION

Malaria is a vector-borne disease caused by a protozoan parasite belonging to the Apicomplexa taxon called *Plasmodium*. This disease is a global health problem, with 247 million cases and 619,000 deaths worldwide in 2021—increasing since 2019 (1). Over the 2 peak years of the pandemic (2020–2021), COVID-related disruptions led to about 13 million more malaria cases and 63, 000 more malaria deaths (1). The diagnosis can be made by microscopy, rapid diagnostic tests, and molecular biology tests. Nevertheless, thin blood smears remain the gold standard technique, enabling species identification and parasitaemia calculation (2). Yet they require highly skilled and trained microscopists and do not prevent frequent misidentification (3). Species misdiagnosis may lead to inappropriate treatment, like chloroquine for *Plasmodium falciparum* or lack of anti-relapse treatment for *P. vivax* and *P. ovale* (4). The lack of microscopic diagnostic skills often gives unacceptable results (5), requiring training sessions (6), which are not always easy to implement.

Another recent approach could be to develop diagnostic tools based on deep learning, aiming at intra-erythrocytic *Plasmodium* detection on images of thick and thin blood smears. These are currently an expanding field of research in the microbiology field (7). The usual process of training neural network algorithms involves several steps: blood smear image acquisition; segmentation of smear images to obtain cell-sized images; and labeling these cell-sized images as infected or uninfected, for example, dividing the database of cell-sized images into an 80% training set and a 20% validation set, training the neural network, and evaluating the performance on a test set consisting of previously unseen images (8).

Many studies (9, 10) aimed to compare different deep learning algorithms, mainly convolutional neural networks (CNN) or their derivates, using publicly available databases such as the National Institute of Health Malaria data set (11) or the Broad Bioimage Benchmark Collection (BBBC) (12). The more frequent drawbacks observed in these studies were the lack of a test data set, with the results calculated only on the validation data set; no patient-level results but only smear images or cell-sized results; too homogenous staining (not reflecting real diversity in routine practice); and issues related with the segmentation process. Segmentation of erythrocytes would be unsuccessful in real-life situations such as overlapping of red blood cells or differences in staining and slide preparation, especially if these cases are not included in the data set (13). To overcome these issues, object detection algorithm-based approaches including mask Regional-convolutional neural Nntwork (R-CNN) (14), faster R-CNN (12), and modified YOLOv3, v4, and v5 algorithms (15, 16) were developed, as they do not need a segmentation step and showed excellent results in terms of accuracy. Among them, YOLO-derived algorithms gave better results than the faster R-CNN (15, 17).

Current state of the art in malaria machine-learning-based detection is the EasyScan GO microscopy device, which reached the field evaluation stage using thick smears (18, 19). The same device was used to develop an algorithm framework including multiple CNNs for thin smears (20) and was evaluated on a 55-slide set from the World Health Organization (WHO) with 82.9% species identification accuracy on both positive and negative samples and 60.0% accuracy on positive samples exclusively (21). Nevertheless, EasyScan Go used x400 magnification images, whereas for precise species identification, microscopists used ×1,000 magnification. Moreover, the EasyScan Go system runtime was 54.4 minutes per slide, which is above the threshold of 10 minutes per slide recommended by the WHO malaria microscopy quality assurance manual (21). Here, to try to overcome the aforementioned issues, a newly available real-time detection transformer (RT-DETR) object detection algorithm was trained and evaluated with a data set made of ×1,000 magnification images taken from thin blood smears. Thus, this study aimed to evaluate and compare the accuracy and performances of the object detection machine-learning algorithm RT-DETR in the species detection of the four main *Plasmodium* species. We also included in our study two less frequently encountered blood parasites: *Trypanosoma brucei* and *Babesia divergens*. The latter is intra-erythrocytic and can be confused with *Plasmodium* spp (22). Moreover, the performances of the RT-DETR algorithm were compared with those of the other two object detection algorithms: YOLOv5 and YOLOv8.

## MATERIALS AND METHODS

### Data collection

The training and validation data set included 24,720 pictures taken from 475 manually May Grunwald–Giemsa (MGG)-stained thin blood smears from the Montpellier University Hospital collection and for a smaller part from the Toulouse University Hospital collection. In Montpellier, the pictures were taken with a FlexCam C1 microscope camera (Leica) attached to a Leica DM 2000 microscope and Leica DF450C microscope camera adapted with a Leica DM2500 microscope at ×1,000 magnification. Labeling of pictures was performed manually and then automatically with manual correction with a Computer Visual Annotation Tools (CVAT) free software. Nine categories of labels were used: white blood cells (*n* = 3,338), red blood cells (*n* = 1,887,781), platelets (*n* = 48,520), *Trypanosoma brucei* (*n* = 2,773), and red blood cells infected by *P. falciparum* (*n* = 43,545), *P. ovale* (*n* = 4,651), *P. vivax* (*n* = 4,115), *P. malariae* (*n* = 2,732), and *Babesia divergens* (*n* = 5,142).

The test data set included 4,508 pictures taken from 170 thin blood smears from the same number of patients from the Parasitology laboratories of University Hospitals of Montpellier, Toulouse, Rouen, Lille, Nantes, and Saint-Louis in Paris (Table 1). Among these 170 patients, 54 were not infected, including two patients with Howell–Jolly bodies, and 116 were infected with hematozoa. For each patient, between 20 and 30 photos were taken from one thin blood smear, with at least one hematozoan parasite per picture for infected patients.

**TABLE 1:** Data set characteristics

Data set	Microscope	Camera	Smear number	Negative smears	*P. falciparum* smears	*P. ovale* smears	*P. malariae* smears	*P. vivax* smears	*Babesia divergens* smears	*Trypanosoma brucei* smears
Training data set			403	57	176	52	48	40	8	22
Montpellier	Leica Flexcam C1 Leica DM 2000	Leica DM2500 Leica Flexcam C1	379	57	171	47	43	35	6	20
Toulouse	Nikon Eclipse E4000	Nikon DS-Fi2	24	0	5	5	5	5	2	2
Validation data set Montpellier	Leica Flexcam C1 Leica DM 2000	Leica DM2500 Leica Flexcam C1	72	19	15	9	5	17	2	5
Test data set			170	54	43	23	22	22	3	3
Lille	Zeiss AXIO Imager.M2 4 slides Fluo/Color		15	0	9	1	1	2	1	1
Montpellier	Leica Flexcam C1	Leica Flexcam C1	65	54	11	0	0	0	0	0
Nantes	Leica DFC 280	Leica DM LB2	1	0	1	0	0	0	0	0
Rouen	Olympus BX41	Olympus SC50	45	0	12	12	11	10	0	0
Saint Louis	Olympus BX41	Olympus DP22	22	0	5	5	5	5	1	1
Toulouse	Nikon Eclipse E4000	Nikon DS-Fi2	22	0	5	5	5	5	1	1

Data set	Image number	Label number	White blood cell labels	Red blood cell labels	Platelet labels	*P. falciparum* labels	*P. ovale* labels	*P. malariae* labels	*P. vivax* labels	*Babesia divergens* labels	*Trypanosoma* *brucei* labels
Train Data set	20830	1677051	2949	1579822	42461	36241	3771	2164	3246	4262	2135
Montpellier	20209	1635166	2921	1540781	40961	35991	3635	2032	3093	3673	2079
Toulouse	621	41885	28	39041	1500	250	136	132	153	589	56
Validation dataset Montpellier	3890	325546	389	307959	6059	7304	880	568	869	880	638
Test dataset	4508	358825	416	342855	10384	2965	696	514	706	221	68
Lille	510	76027	24	73735	1222	665	52	33	187	77	32
Montpellier	2034	124226	243	117812	5440	684	1	0	46	0	0
Nantes	25	2882	0	2671	90	121	0	0	0	0	0
Rouen	981	107767	92	103148	2431	1269	263	279	285	0	0
Saint Louis	425	13570	33	12842	282	22	252	78	45	1	15
Toulouse	533	34353	24	32647	919	204	128	124	143	143	21

Accurate species diagnosis was made by a senior parasitologist, and for recent smears, it was confirmed by specific PCR, either performed locally (Toulouse) or at the Malaria French National Reference Center (Montpellier, Saint Louis, Rouen, Lille, Nantes).

### Algorithm training and validation

The training and validation data sets were split into 80% training and 20% validation to train the real-time object detection neural network algorithms RT-DETR (https://arxiv.org/abs/2304.08069), YOLOv5x (https://github.com/ultralytics/yolov5), and YOLOv8x (https://github.com/ultralytics/ultralytics) using the Pytorch framework. Images from the same patient contributed to either the training or validation data set, but not to both, to avoid bias. Parameters of algorithm training were as follows: image resolution 640 pp, epoch 25, patience 5, and batch size 32. The models were trained on an NVIDIA GeForce RTx3060 GPU with 12 GB of memory in 13.106 hours (14 epochs) for the RT-DETR model, in 14.976 hours (25 epochs) for the YOLOv5x model, and in 10.384 hours (23 epochs) for the YOLOv8x model. Overfitting was not observed during training of the models (Fig. S1 through S3).

### Algorithm testing

The test data set images were resized at 16:9, and a zoom correction was applied to fit the images of the train/validation data set. Performances of the models were assessed at the label and patient level, thanks to the multicentric test data set. The label level corresponds to the raw data obtained directly from the object detection algorithm. Indeed, the object detection models work on a frame-by-frame basis. In each image, around a hundred objects are labeled, corresponding to uninfected red blood cells, platelets, and red blood cells infected by various parasites. The patient-level detection comes after this initial detection stage and consists of compiling the labels obtained from several images belonging to the same smear to give a general result: uninfected, infected by this, or infected by that species.

At the label level, the performances for each label category were evaluated with precision = $\frac{TP}{TP+FP}$with TP, true positive and FP, false positive; recall = $\frac{TP}{TP+FN}$ with FN, false negative; F1 score = $\frac{2\left(precisionxrecall\right)}{precision+recall}$ ; Matthews correlation coefficient = $\frac{\left(TN*TP-FP*FN\right)}{\sqrt{\left(TN+FN)(FP+TP)(TN+FP)(FN+TP\right)}}$ with TN, true negative ; mean average precision at an intersection over union (IoU) of 0.5 (mAP@.5) and average mAP over different IoU thresholds, from 0.5 to 0.95, step 0.05 (mAP@.5:.95) (23) (Table 2).

**TABLE 2:** Overall and per class results of the test dataset with the RT-DETR model^*a*^

Class	Images	Labels	Precision	Recall	mAP@.5	mAP@.5:.95:	F1 score	MCC
All	4508	275240	0.686	0.669	0.638	0.596	0.677	0.633
WBC	4508	363	0.798	0.953	0.956	0.904	0.869	0.855
RBC	4508	261789	0.973	0.992	0.994	0.976	0.982	0.723
Platelets	4508	8581	0.895	0.956	0.979	0.9	0.924	0.852
*P. falciparum*	4508	2488	0.819	0.845	0.858	0.845	0.832	0.827
*P. ovale*	4508	530	0.417	0.353	0.199	0.193	0.382	0.38
*P. malariae*	4508	500	0.679	0.604	0.644	0.626	0.639	0.637
*P. vivax*	4508	672	0.376	0.284	0.15	0.143	0.324	0.326
*Babesia*	4508	250	0.385	0.32	0.203	0.197	0.35	0.34
*Trypanosoma brucei*	4508	67	0.827	0.716	0.764	0.579	0.768	0.756

^*a*^ MCC: Matthews correlation coefficient.

**Fig 1:**
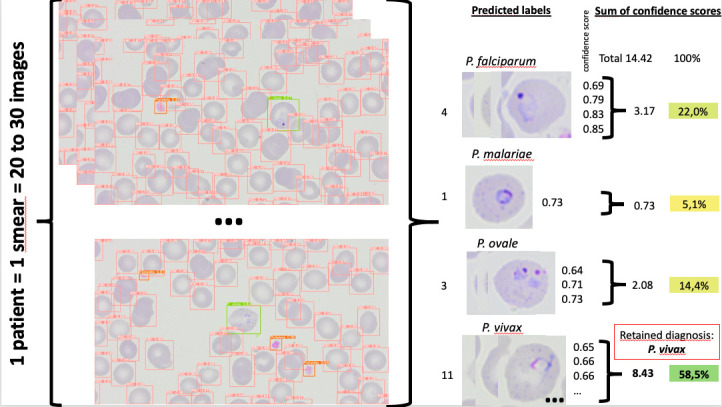
Determination of parasitic diagnosis at the patient level. Example of a smear from the test data set. *P. vivax* was selected as the final diagnosis because its sum of confidence scores is the highest (8.43 > 3.17 > 2.08 > 0.73).

At the patient level, the label confidence scores of each parasite were summed over the 20 to 30 pictures (Fig. 1), and the parasite with the highest sum of confidence scores was selected as the final diagnosis. Each label had a confidence score between 0 and 1. Results at patient level were presented in the form of a confusion matrix (Table 3) and were assessed in terms of accuracy (true predictions/ all predictions), precision, and recall.

**TABLE 3:** Results by class of the test data set at the patient level with the RT-DETR model^*a*^^,^^*b*^

Predicted / True	*Babesia*	*P. falciparum*	*P. malariae*	*P. ovale*	*P. vivax*	Not infected	*Trypanosoma brucei*	Precision
*Babesia*	1	2			1			**0.25**
*P. falciparum*	1	38	4	2	8	3		**0.68**
*P. malariae*		1	18			10		**0.62**
*P. ovale*				10	8	1		**0.53**
*P. vivax*	1	1		11	6			**0.32**
Not infected						40		**1**
*Trypanosoma brucei*							3	**1**
**Recall**	**0.33**	**0.90**	**0.82**	**0.43**	**0.26**	**0.74**	**1**	
**F1 score**	**0.29**	**0.78**	**0.71**	**0.48**	**0.29**	**0.85**	**1**	
**MCC**	**0.27**	**0.70**	**0.66**	**0.41**	**0.19**	**0.81**	**1**	

^*a*^ MCC: Matthews correlation coefficient.

^*b*^ Figures in bold refer to metrics calculated from non-bold figures.

## RESULTS

### Labels level

**Fig 2:**
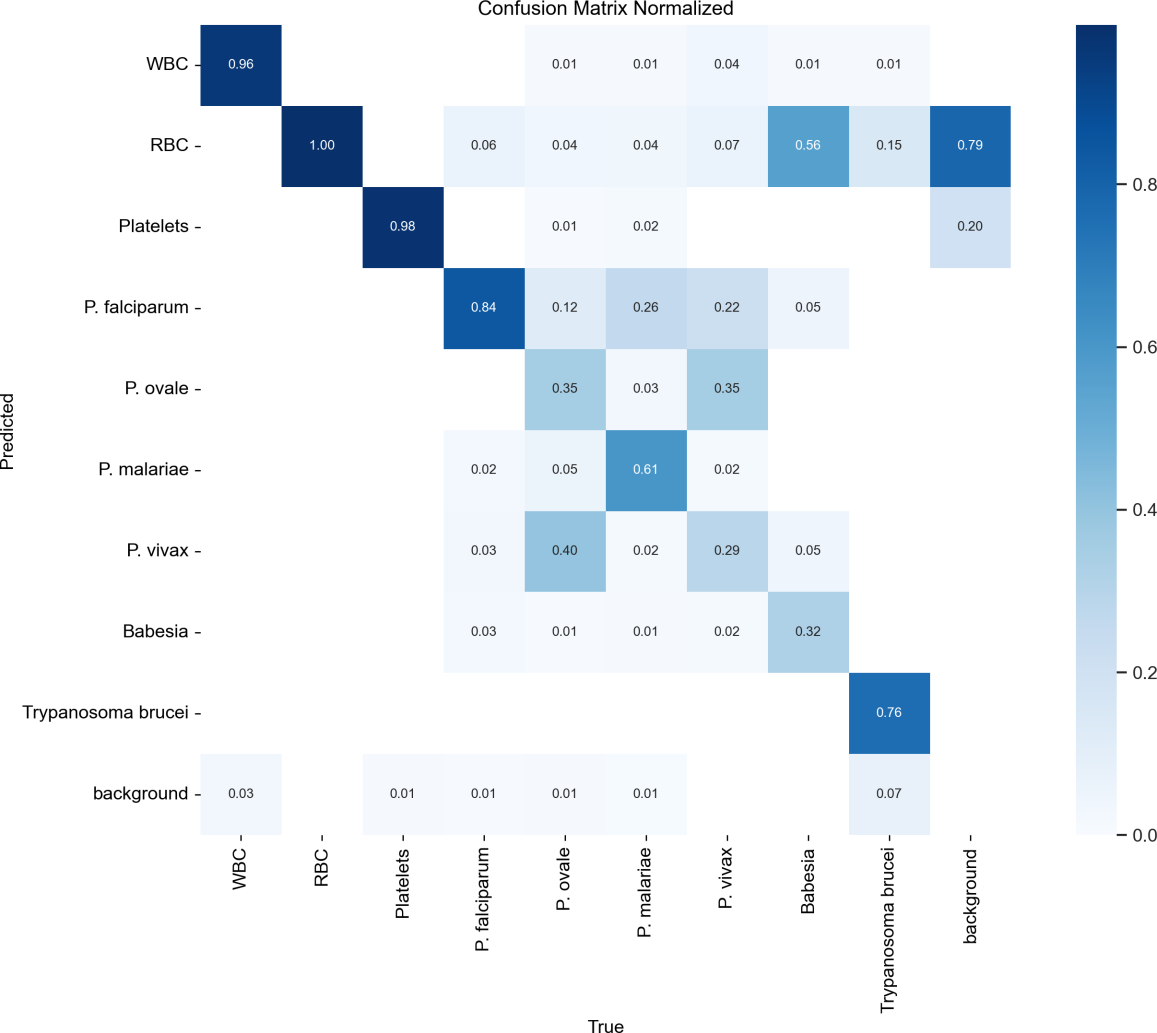
Confusion matrix with test data set labels of the RT-DETR model.

Out of the 4,508 images of the 170 smears in the test data set, 358,825 labels were generated by the model. At the label level, overall precision, recall, and mAP@.5 were 0.686, 0.669, and 0.638, respectively (Table 2). However, performance varied greatly by class. The mAP@.5 was greater than 0.95 for white blood cells, red blood cells, and platelets. The mAP@.5 was 0.858 for *P. falciparum*, 0.764 for *Trypanosoma brucei,* and 0.644 for *P. malariae*. This metric was lower for *P. ovale* (0.199) and *P. vivax* (0.15). The confusion matrix (Fig. 2) showed a significant mislabeling between the latter two parasites since 40% of the *P. ovale* were labeled as *P. vivax* and 35% of the *P. vivax* were labeled as *P. ovale*. The model poorly performed in detecting *Babesia divergens* parasites, with a precision of 0.385 and a recall of 0.32.

Parameters used for the confusion matrix were as follows: confidence score threshold equal to or greater than 0.25; IoU equal to or greater than 0.45; agnostic = True.

### Patient level

At the patient level, the test data set included 170 thin blood smears from 170 patients, of which 54 were uninfected and 116 infected with either *P. falciparum, P. malariae*, *P. ovale*, *P. vivax*, *Babesia divergens,* or *Trypanosoma brucei* (Table 3). The overall accuracy with the six parasite class RT-DETR model was 68.2% (116/170). The recall was 100% (3/3) for *Trypansoma brucei*, 90% (38/42) for *P. falciparum*, 74% (40/54) for negative smears, and 81.8% (18/22) for *P. malariae*. The recall was lower for *P. vivax* (26%, 6/23), *P. ovale* (43%, 10/23), and *Babesia divergens* (33%, 1/3). Misdiagnosis between *P. ovale* and *P. vivax* was common, and among the 46 *P*. *ovale/vivax* smears, eight (17.4%) were *P. vivax* diagnosed as *P. ovale a*nd 11 (23.9%) were *P. ovale* diagnosed as *P. vivax*. Taking this into account, a five-parasite class model gathering *P. ovale* and *P. vivax* labels showed an overall accuracy of 79.4% (135/170) with a recall of 76.1% for the *P. ovale/vivax* class (35/46). For the classification of patients into infected and uninfected, the accuracy was 91.8% (156/170).

### Algorithm comparison

The performance of the RT-DETR model was compared with that of the YOLOv5x model and of the YOLOv8x model (Table 4). At the label level, YOLOv8x seems to be slightly better than the other models, with an mAP@.5 of 0.727 (Fig. S14) vs 0.67 for the YOLOv5x (Fig. S25) and 0.638 for RT-DETR (Table 2). At the patient level, the overall accuracy of the three models was similar: 62.8% (116/170) for the RT-DETR and the YOLOv5x model (Table S3) and 67% (114/170) for the YOLOv8x model (Table S4).

**TABLE 4:** Comparison of patient-level results of three algorithm models^*a*^

		YOLOv5x	YOLOv8x	RT-DETR
Accuracy, number of	Six-parasite class model	116 (68.2)	114 (67)	116 (68.2)
Correct diagnosis /170 (%)	Five-parasite class model	137 (80.6)	129 (75.3)	135 (79.4)
	Four-parasite class model	139 (81.8)	137 (80.6)	135 (79.4)
	Two-parasite class model	160 (94.1)	160 (94.1)	156 (91.8)
Accuracy, number of	Four-*Plasmodium* class model	66 (60)	66 (60)	72 (65.5)
Correct diagnosis /110 (%)	Three-*Plasmodium* class model	87 (79.1)	81 (73.6)	91 (82.7)
	Two-*Plasmodium* class model	89 (81)	89 (81)	91 (82.7)
*Babesia divergens*	Precision	0	0.33	0.25
	Recall	0	0.33	0.33
	F1-score	0	0.33	0.29
*P. falciparum*	Precision	0.63	0.73	0.68
	Recall	0.86	0.69	0.90
	F1-score	0.73	0.71	0.78
*P. malariae*	Precision	0.93	0.61	0.62
	Recall	0.59	0.5	0.82
	F1-score	0.72	0.55	0.71
*P. ovale*	Precision	0.40	0.41	0.53
	Recall	0.74	0.91	0.43
	F1-score	0.52	0.57	0.48
*P. vivax*	Precision	0	0.56	0.32
	Recall	0	0.21	0.26
	F1-score	0	0.31	0.29
Not infected	Precision	0.98	1	1
	Recall	1	0.81	0.74
	F1-score	0.9	0.90	0.85
*Trypanosoma brucei*	Precision	0.6	0.69	1
	Recall	1	1	1
	F1-score	0.75	0.75	1

^*a*^ Six-parasite class model (*T. brucei, B. divergens, P. falciparum, P. malariae, P. ovale,* and *P. vivax,* none infected). Five-parasite class model (*T. brucei, B. divergens, P. falciparum, P. malariae,* and *P. ovale/vivax*, none infected). Four-parasite class model (*T. brucei, B. divergens, P. falciparum,* and *P. malariae/ovale/vivax*, none infected). Two-parasite class model (*T. brucei*, intraerythrocytic parasites, none infected). Four-*Plasmodium* class model (*P. falciparum, P. malariae, P. ovale,* and *P. vivax*). Three-*Plasmodium* class model (*P. falciparum, P. malariae,* and *P. ovale/vivax*). Two-*Plasmodium* classe model (*P. falciparum* and *P. malariae/ovale/vivax*).

## DISCUSSION

To our knowledge, the RT-DETR model is the first algorithm aiming to identify five hematozoan parasites on thin blood smear images. With a large test data set of 170 thin blood smears from six different centers, the six-parasite class model exhibited an overall accuracy of 68.2% (116/170), while the five-parasite class model, which combines *P. ovale* and *P. vivax,* showed an overall accuracy of 79.4% (135/170) at the patient level.

In respect to the results by parasite class at the patient level, the recall for *Trypanosoma brucei* was 100% (3/3), which was expected due to its easily identifiable extra-erythrocytic nature. The recall for *P. falciparum* was 90% (38/42). Four *P. falciparum* smears were misdiagnosed as *P. malariae* (one smear), *P. vivax* (one smear), and *B. divergens* (two smears). Old trophozoites with Maurer’s clefts were particularly prone to being misdiagnosed as a non-*falciparum* species.

The recall for *P. ovale* and *P. vivax* was 43% (10/23) and 26% (6/23), respectively, showing a high rate of confusion between these two species. Indeed, 19 out of 46 *P*. *vivax/ovale* smears were misdiagnosed as *P. vivax* for *P. ovale* or vice versa. This is coherent with the difficulties encountered by microscopists in routine practice to distinguish both species without geographic priors or rapid diagnostic test information (3). Nevertheless, this error has no therapeutic or clinical impact on patients since the treatment is similar for both species. Thus, grouping *P. ovale* and *P. vivax* in a single class achieved a recall of 76.1% (35/46).

The recall for *P. malariae* was 81.8% (18/22). Four *P. malariae* smears were misclassified as *P. falciparum* due to a high rate of young ring-form trophozoites. A new class could have been trained for *P. malariae* late parasitic stages (equatorial-like bands; rosette schizonts) which are species-specific patterns, but the number of corresponding labels was too small in the data set.

The recall for *Babesia divergens* was only 33% (1/3)%. This may be due to the lack of *Babesia* smears included in the train/validation data set. The recall of negative smears was 74% (40/54). Forty-one out of 100,708 labels (0.04%) corresponding to 14 out of 54 negative smears (25.9%) of the test data set were misclassified as positive. Ten of them were mislabeled as *P. malariae*, three as *P. falciparum,* and one as *P. ovale* (Fig. S5).

A previous study (21) using the EasyScan GO device showed 60% accuracy in identifying the four major *Plasmodium* species on a set of 15 positive smears. This is to be compared with the presented RT-DETR model, which exhibited an overall accuracy of 65.5% (72/110) on 110 *Plasmodium-*positive smears and an 82.7% (91/110) accuracy with the model pooling *P. ovale* and *P. vivax*. The latter model achieved a WHO competence level 2 for species identification (24). For species identification, the presented algorithm could be of great help for inexperienced microscopists and shows advantages over both loop-mediated isothermal amplification (LAMP), which does not discriminate between *Plasmodium* species, and rapid diagnostic tests, which have a very low sensitivity for *P. malariae* and *P. ovale*.

YOLO was described as more performant than other object detection algorithms (R-CNN, SSD) using publicly available malaria data set (15, 17), and RT-DETR was found more performant than YOLO models on the COCO (Common Objects in Context) data set (https://arxiv.org/abs/2304.08069). The RT-DETR algorithm gets rid of the computationally expensive segmentation step used with the CNN to obtain multiple cropped images with a unique red blood cell from a microscope field of view image. Besides, the RT-DETR algorithm can be run in real-time on low-cost devices such as a smartphone with a microscope adapter (25). Such a system could be adapted to improve parasite detection for inexperienced microscopists and to alert them to forms suspected of being *Plasmodium* parasites. A later step will therefore require adapting this RT-DETR algorithm to a device that can be used in the field. Mobile applications have been developed using either CNN, R-CNN such as Malaria Screener (26), or PlasmoCount (27). Beyond manual microscopy, other systems could be considered to reach partial or full automatization such as a robotic handed microscope (28) or hardware slide scan (29).

This study acknowledged some limitations. It was not designed to assess parasite detection and quantification performance of the RT-DETR model but only for species identification purposes. Parasitemia assessment could not be done on our test data set since the images were not captured randomly but focused on parasite forms. Although the test data set included images from six different parasitology laboratories, previously unseen staining artifacts are likely to induce algorithm errors. As for species identification, the parasitic stage should also be considered. Indeed, although late-stage trophozoites, schizonts, and gametocytes are more specific for species identification, they are less abundant than ring and young trophozoites. For this purpose of parasite stage recognition, an unsupervised machine learning approach might be interesting (30). Besides, performances might be improved by coupling the RT-DETR algorithm with expert rules including geographic priors, parasitemia, and rapid diagnostic tests results.

To our knowledge, this study is the first attempt to predict malaria species from thin blood smear images using an RT-DETR object detection algorithm. This algorithm allows accurate real-time detection of parasites on easily affordable devices as smartphones mounted on a microscope. Further studies should aim to develop and evaluate this cell phone application, particularly in malaria endemic countries where trained microscopists are not in sufficient numbers.

## Data Availability

Data and models are available on Zenodo (DOI: 10.5281/zenodo.8358829).

## SUPPLEMENTAL MATERIAL

Supplemental Figures spectrum.01440-23-s0001.docx Figures S1 to S6.

Supplemental Tables spectrum.01440-23-s0002.docx Tables S1 to S5.

ASM does not own the copyrights to Supplemental Material that may be linked to, or accessed through, an article. The authors have granted ASM a non-exclusive, world-wide license to publish the Supplemental Material files. Please contact the corresponding author directly for reuse.
