# Supplementary material for: Automatic patient-level recognition of four Plasmodium species on thin blood smear by a real-time detection transformer (RT-DETR) object detection algorithm: a proof-of-concept and evaluation
Source: Microbiol Spectr. 2024 Jan 3;12(2):e01440-23. doi: 10.1128/spectrum.01440-23 (PMC10846087; doi:10.1128/spectrum.01440-23)
Supplement: Supplemental Figures — Figures S1 to S6. [file spectrum.01440-23-s0001.docx]

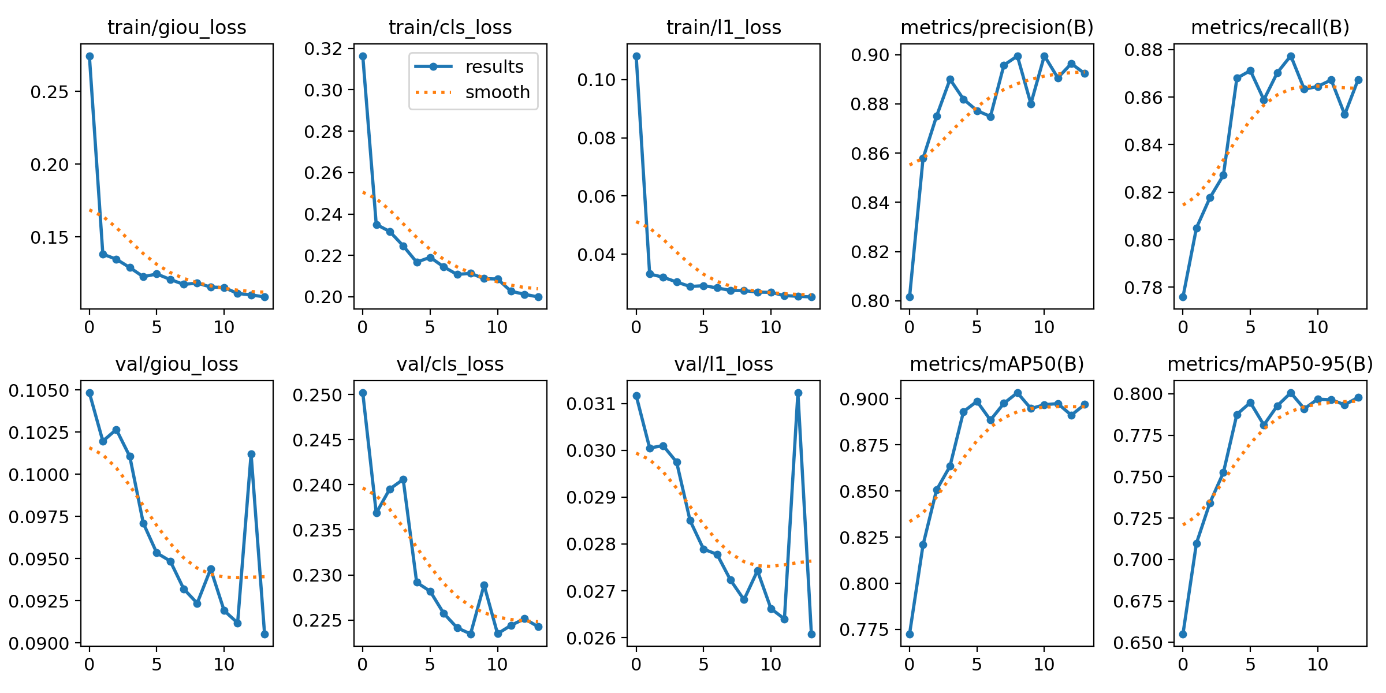


**Figure S1:** Loss and others metrics according to the number of epochs during the training/validation of the RT-DETR model. Train/box_loss (bounding box regression loss during training), train/obj_loss (the confidence of the object presence = objectness loss during training), train/cls_loss (the classification loss during training), val/box_loss (bounding box regression loss during validation), val/obj_loss (the confidence of the object presence = objectness loss during validation), val/cls_loss (the classification loss during training), mAP_0.5 (mean average precision at IoU (Intersection over Union) threshold of 0.5, mAP_0.5:0.95 (average mAP over different IoU thresholds, ranging from 0.5 to 0.95).


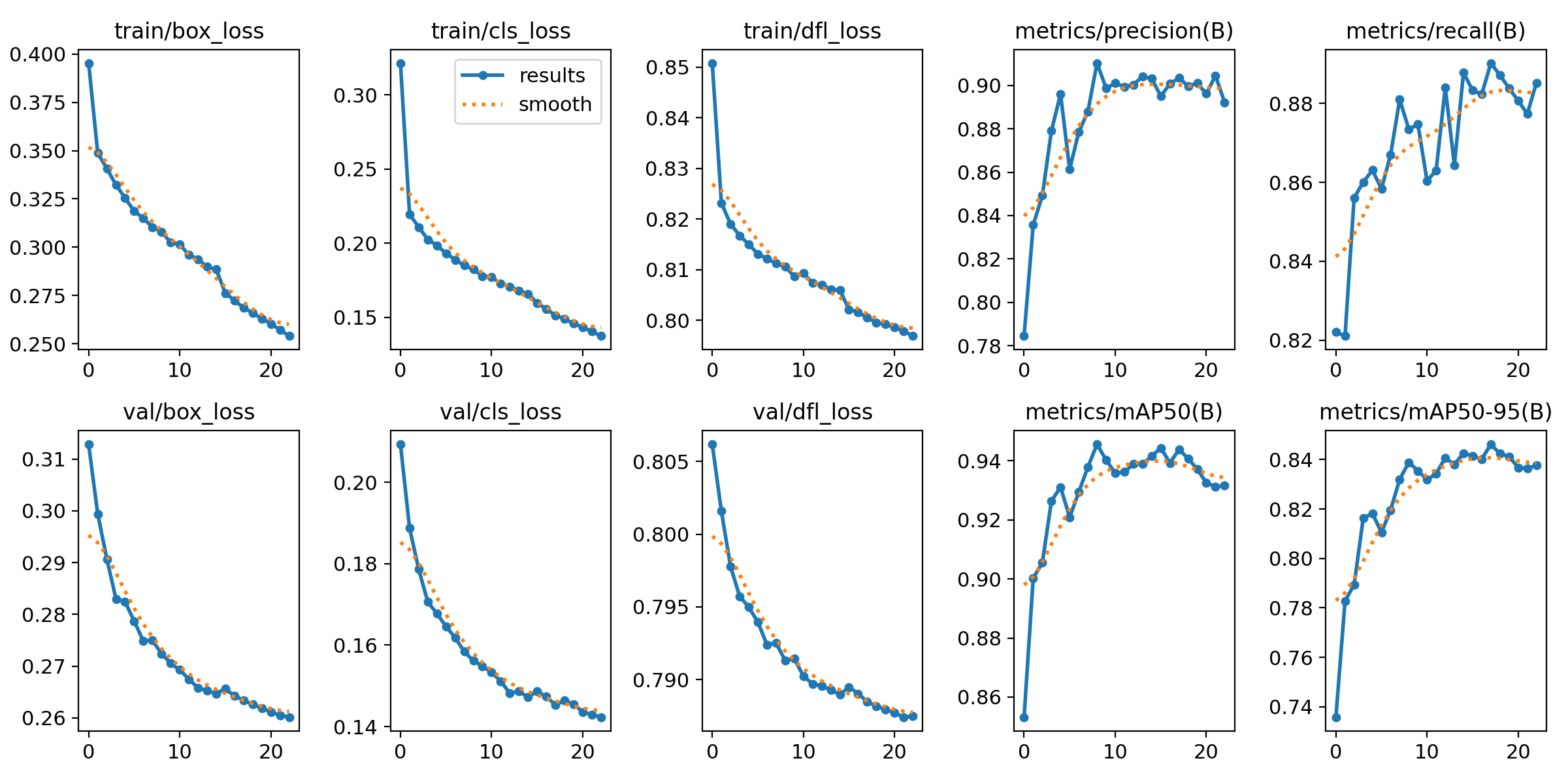


**Figure S2:** Loss and others metrics according to the number of epochs during the training/validation of the YOLOv8 model. Train/box_loss (bounding box regression loss during training), train/obj_loss (the confidence of the object presence = objectness loss during training), train/cls_loss (the classification loss during training), val/box_loss (bounding box regression loss during validation), val/obj_loss (the confidence of the object presence = objectness loss during validation), val/cls_loss (the classification loss during training), mAP_0.5 (mean average precision at IoU (Intersection over Union) threshold of 0.5, mAP_0.5:0.95 (average mAP over different IoU thresholds, ranging from 0.5 to 0.95).


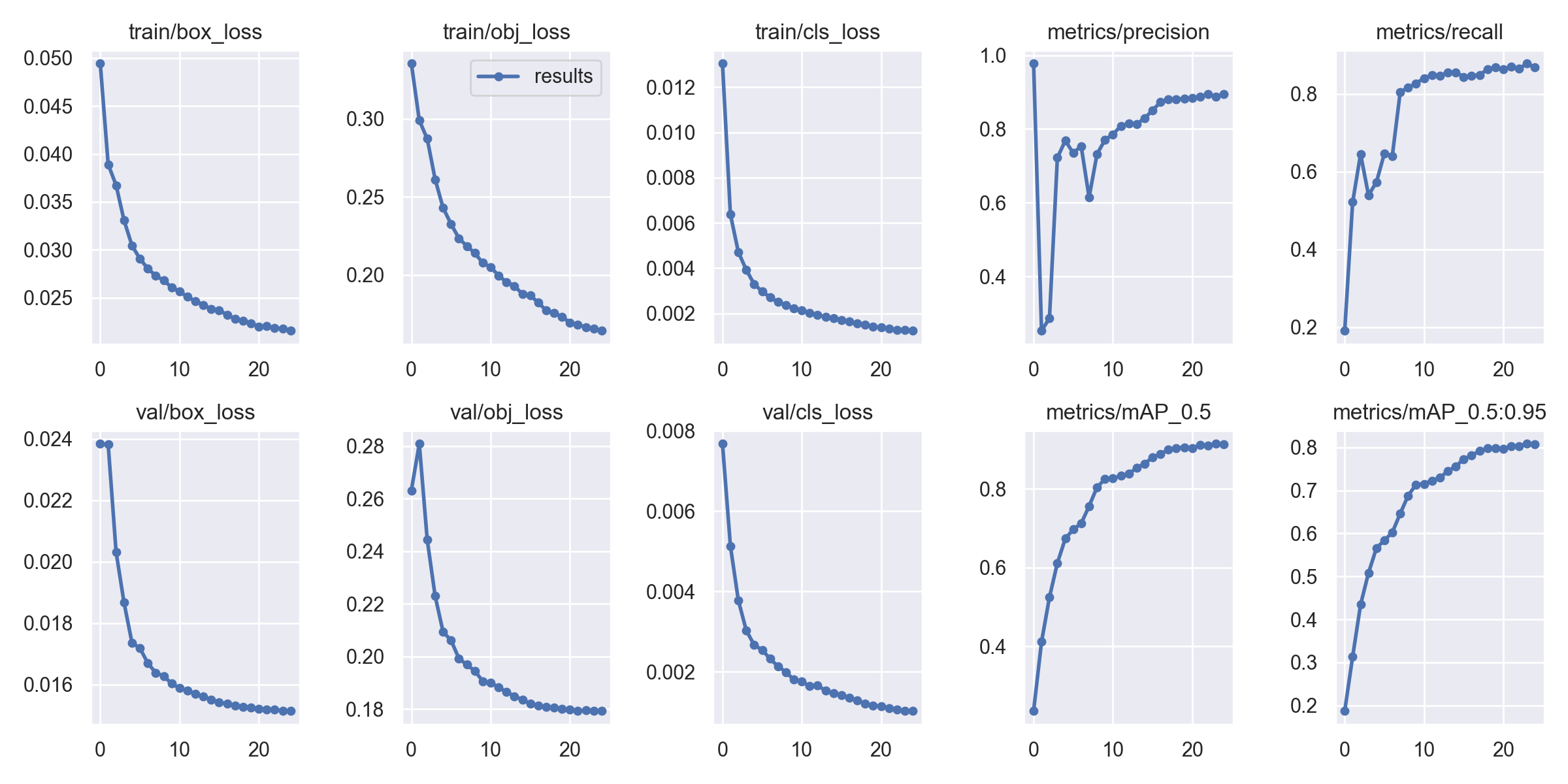


**Figure S3:** Loss and others metrics according to the number of epochs during the training/validation of the YOLOv5 model. Train/box_loss (bounding box regression loss during training), train/obj_loss (the confidence of the object presence = objectness loss during training), train/cls_loss (the classification loss during training), val/box_loss (bounding box regression loss during validation), val/obj_loss (the confidence of the object presence = objectness loss during validation), val/cls_loss (the classification loss during training), mAP_0.5 (mean average precision at IoU (Intersection over Union) threshold of 0.5, mAP_0.5:0.95 (average mAP over different IoU thresholds, ranging from 0.5 to 0.95).


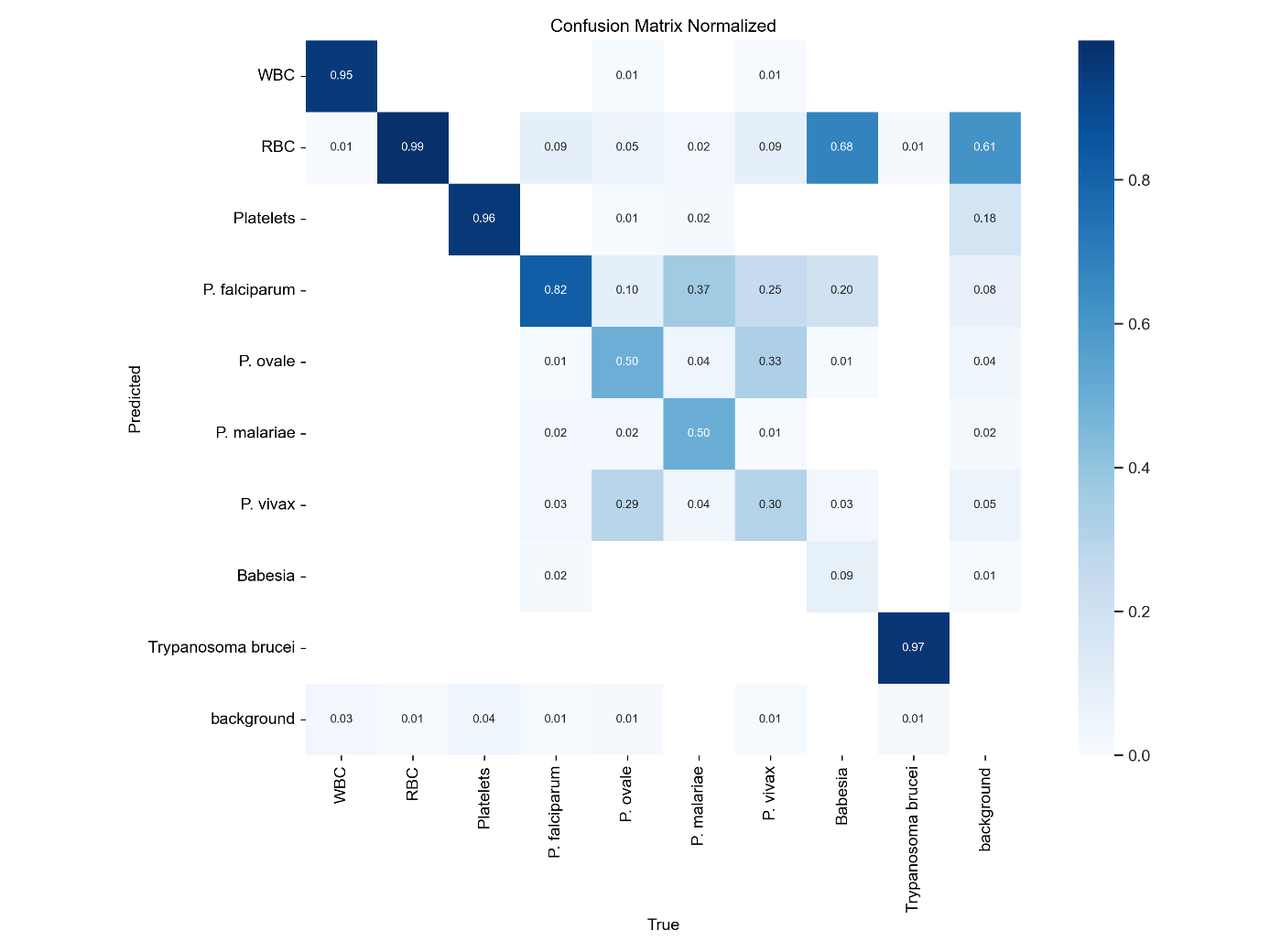


**Figure S4:** Confusion matrix with test dataset labels of the YOLOv8 model. Parameters used for the confusion matrix were the following: confidence score threshold equal to or greater than 0.25, IoU equal to or greater than 0.45, agnostic=True.


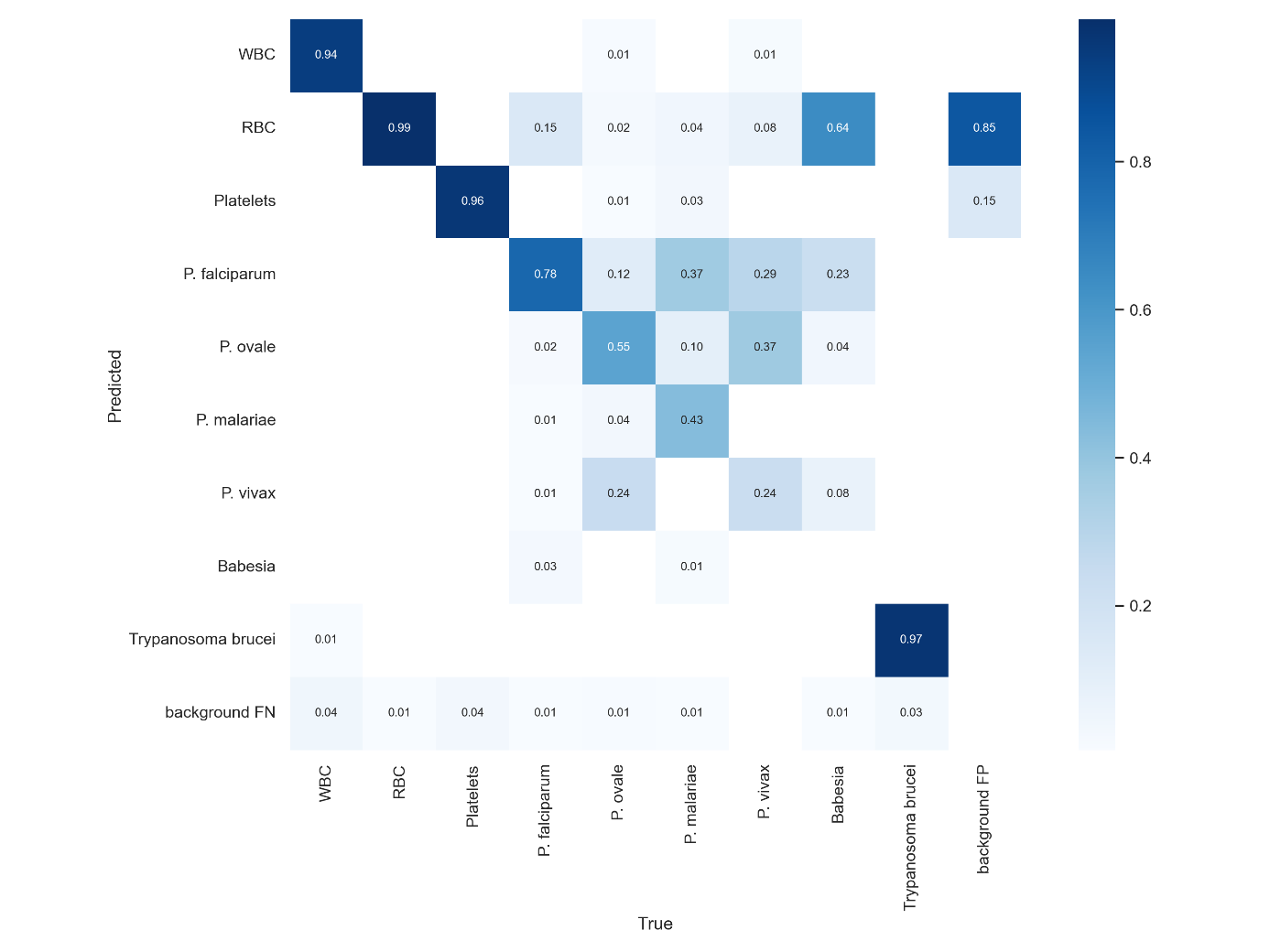


**Figure S5:** Confusion matrix with test dataset labels of the YOLOv5 model. Parameters used for the confusion matrix were the following: confidence score threshold equal to or greater than 0.25, IoU equal to or greater than 0.45, agnostic=True.


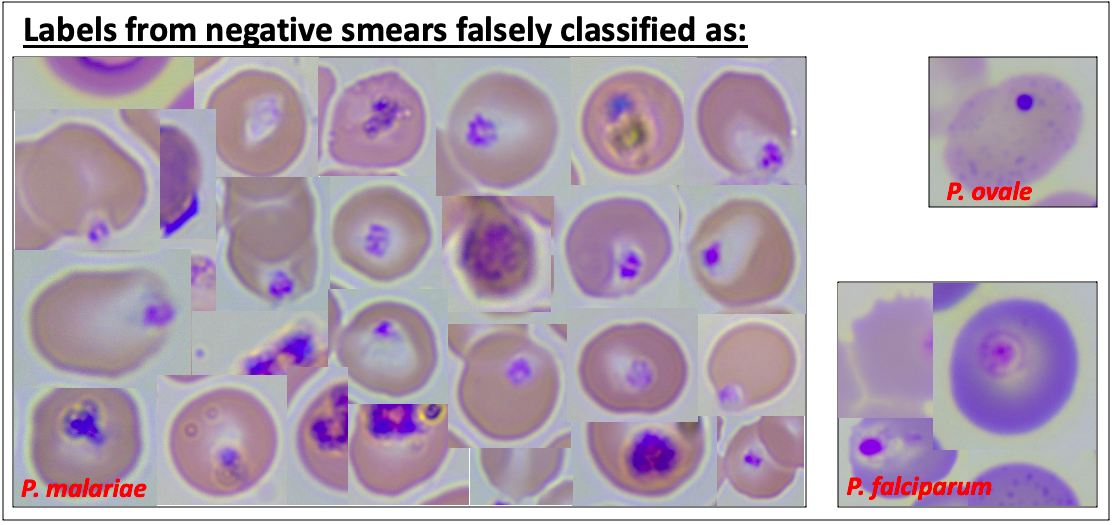


**Figure S6***:* Labels from negative smears falsely classified as parasites
