# Supplementary material for: Automatic patient-level recognition of four Plasmodium species on thin blood smear by a real-time detection transformer (RT-DETR) object detection algorithm: a proof-of-concept and evaluation
Source: Microbiol Spectr. 2024 Jan 3;12(2):e01440-23. doi: 10.1128/spectrum.01440-23 (PMC10846087; doi:10.1128/spectrum.01440-23)
Supplement: Supplemental Tables — Tables S1 to S5. [file spectrum.01440-23-s0002.docx]

| **Class** | **Images** | **Labels** | **Precision** | **Recall** | [**mAP@.5**](mailto:mAP@.5) | [**mAP@.5:.95:**](mailto:mAP@.5:.95:) | **F1 score** | **MCC** |
| --- | --- | --- | --- | --- | --- | --- | --- | --- |
| **all** | 4508 | 275240 | 0.699 | 0.734 | 0.727 | 0.697 | 0.716 | 0.638 |
| **WBC** | 4508 | 363 | 0.914 | 0.956 | 0.967 | 0.933 | 0.934 | 0.933 |
| **RBC** | 4508 | 261789 | 0.988 | 0.994 | 0.995 | 0.989 | 0.991 | 0.85 |
| **Platelets** | 4508 | 8581 | 0.909 | 0.961 | 0.984 | 0.937 | 0.934 | 0.933 |
| ***P. falciparum*** | 4508 | 2488 | 0.757 | 0.883 | 0.897 | 0.890 | 0.815 | 0.756 |
| ***P. ovale*** | 4508 | 530 | 0.499 | 0.669 | 0.470 | 0.466 | 0.572 | 0.429 |
| ***P. malariae*** | 4508 | 500 | 0.703 | 0.576 | 0.704 | 0.683 | 0.633 | 0.553 |
| ***P. vivax*** | 4508 | 672 | 0.421 | 0.439 | 0.403 | 0.399 | 0.43 | 0.296 |
| ***Babesia*** | 4508 | 250 | 0.343 | 0.152 | 0.156 | 0.154 | 0.211 | 0.133 |
| ***Trypanosoma brucei*** | 4508 | 67 | 0.757 | 0.979 | 0.970 | 0.818 | 0.854 | 0.856 |

**Table S1:** Overall and per class results of the test dataset with the YOLOv8 model. MCC (Matthews correlation coefficient).

| **Class** | **Images** | **Labels** | **Precision** | **Recall** | [**mAP@.5**](mailto:mAP@.5) | [**mAP@.5:.95:**](mailto:mAP@.5:.95:) | **F1 score** | **MCC** |
| --- | --- | --- | --- | --- | --- | --- | --- | --- |
| **all** | 4508 | 275240 | 0.71 | 0.648 | 0.67 | 0.647 | 0.678 | 0.647 |
| **WBC** | 4508 | 363 | 0.938 | 0.937 | 0.961 | 0.943 | 0.937 | 0.934 |
| **RBC** | 4508 | 261789 | 0.992 | 0.99 | 0.994 | 0.987 | 0.991 | 0.801 |
| **Platelets** | 4508 | 8581 | 0.953 | 0.944 | 0.977 | 0.927 | 0.948 | 0.939 |
| ***P. falciparum*** | 4508 | 2488 | 0.761 | 0.78 | 0.785 | 0.779 | 0.77 | 0.767 |
| ***P. ovale*** | 4508 | 530 | 0.449 | 0.549 | 0.364 | 0.358 | 0.494 | 0.492 |
| ***P. malariae*** | 4508 | 500 | 0.87 | 0.43 | 0.659 | 0.645 | 0.576 | 0.611 |
| ***P. vivax*** | 4508 | 672 | 0.498 | 0.236 | 0.316 | 0.314 | 0.32 | 0.341 |
| ***Babesia*** | 4508 | 250 | 0 | 0 | 0 | 0 | 0 | 0 |
| ***Trypanosoma brucei*** | 4508 | 67 | 0.924 | 0.97 | 0.975 | 0.874 | 0.946 | 0.942 |

**Table S2:** Overall and per class results of the test dataset with the YOLOv5 model. MCC (Matthews correlation coefficient).

| **True   Predicted** | *Babesia* | *P. falciparum* | *P. malariae* | *P. ovale* | *P. vivax* | Not infected | *Trypanosoma brucei* | **Precision** |
| --- | --- | --- | --- | --- | --- | --- | --- | --- |
| *Babesia* |  | 1 |  |  |  |  |  | **0** |
| *P. falciparum* | 2 | **36** | 7 | 1 | 7 | 4 |  | **0.63** |
| *P. malariae* |  |  | **13** |  |  | 1 |  | **0.93** |
| *P. ovale* | 1 | 4 | 2 | **17** | 16 | 2 |  | **0.40** |
| *P. vivax* |  |  |  | 5 |  |  |  | **0** |
| Not infected |  | 1 |  |  |  | **45** |  | **0.98** |
| *Trypanosoma brucei* |  |  |  |  |  | 2 | **3** | **0.6** |
| **Recall** | **0** | **0.86** | **0.59** | **0.74** | **0** | **0.83** | **1** |  |
| **F1 score** | **0** | **0.73** | **0.72** | **0.52** | **0** | **0.9** | **0.75** |  |
| **MCC** | **0** | **0.63** | **0.71** | **0.45** | **0** | **0.86** | **0.77** |  |

**Table S3:** Results by class of the test dataset at the patient level with the YOLOv5 model. MCC (Matthews correlation coefficient)

| **True  Predicted** | *Babesia* | *P. falciparum* | *P. malariae* | *P. ovale* | *P. vivax* | Not infected | *Trypanosoma brucei* | **Precision** |
| --- | --- | --- | --- | --- | --- | --- | --- | --- |
| *Babesia* | **1** | 2 |  |  |  |  |  | **0.33** |
| *P. falciparum* | 1 | **29** | 4 |  | 4 | 2 |  | **0.73** |
| *P. malariae* |  | 1 | **11** |  | 1 | 5 |  | **0.61** |
| *P. ovale* | 1 | 8 | 7 | **21** | 13 | 1 |  | **0.41** |
| *P. vivax* |  | 2 |  | 2 | **5** |  |  | **0.56** |
| Not infected |  |  |  |  |  | **44** |  | **1** |
| *Trypanosoma brucei* |  |  |  |  |  | 2 | **3** | **0.6** |
| **Recall** | **0.33** | **0.69** | **0.5** | **0.91** | **0.21** | **0.81** | **1** |  |
| **F1 score** | **0.33** | **0.71** | **0.55** | **0.57** | **0.31** | **0.90** | **0.75** |  |
| **MCC** | **0.32** | **0.61** | **0.49** | **0.53** | **0.29** | **0.87** | **0.77** |  |

**Table S4:** Results by class of the test dataset at the patient level with the YOLOv8 model. MCC (Matthews correlation coefficient)

| **Smear name** | **Origin** | ***Babesia divergens*** | | ***P. falciparum*** | | ***P. malariae*** | | ***P. ovale*** | | ***P. vivax*** | | **Platelets** | | **Red Blood Cells** | | ***Trypanosoma brucei*** | | **White Blood Cells** | | **Total of labels** | | **True species** | **Predicted species** | **Correct prediction ?** |
| --- | --- | --- | --- | --- | --- | --- | --- | --- | --- | --- | --- | --- | --- | --- | --- | --- | --- | --- | --- | --- | --- | --- | --- | --- |
|  |  | **sum of score  confidence** | **labels count** | **sum of score  confidence** | **labels count** | **sum of score  confidence** | **labels count** | **sum of score  confidence** | **labels count** | **sum of score  confidence** | **labels count** | **sum of score  confidence** | **labels count** | **sum of score  confidence** | **labels count** | **sum of score  confidence** | **labels count** | **sum of score  confidence** | **labels count** | **sum of score  confidence** | **labels count** |  |  |  |
| afbda56e-b471-40fe-addc-3f3fe8e862be | Lille |  |  | 5.443872 | 8 |  |  |  |  |  |  | 0.63245 | 1 | 2092.667457 | 2212 |  |  |  |  | 2098.743779 | 2221 | B. divergens | P. falciparum | no |
| 197dd3d4-deaa-4769-a7df-3bcb04f71026 | Toulouse | 59.221073 | 79 | 2.294071 | 4 |  |  |  |  |  |  | 6.374103 | 8 | 586.620822 | 649 |  |  | 1.770819 | 2 | 656.280888 | 742 | B. divergens | B. divergens | yes |
| b35d8716-1578-4f42-9102-d2d955b0cfe7 | Saint-Louis |  |  |  |  |  |  |  |  | 7.165061 | 11 | 38.883877 | 52 | 711.245324999999 | 758 |  |  | 1.845213 | 2 | 759.139475999999 | 823 | B. divergens | P. vivax | no |
| 8fb66df2-7bf8-4ac5-a06c-169648d92fd5 | Montpellier |  |  |  |  |  |  |  |  |  |  | 134.749281 | 150 | 1357.322692 | 1398 |  |  | 1.939887 | 2 | 1494.01186 | 1550 | negative | negative | yes |
| b1db458d-9551-48bd-b79e-a93c93b70ade | Montpellier |  |  |  |  |  |  |  |  |  |  | 21.933236 | 27 | 2369.960842 | 2465 |  |  | 3.40198 | 4 | 2395.296058 | 2496 | negative | negative | yes |
| 404a4dda-68d4-450a-b856-24f07bc3095b | Montpellier |  |  |  |  |  |  |  |  |  |  | 28.548791 | 37 | 2544.681738 | 2623 |  |  | 0.823707 | 1 | 2574.054236 | 2661 | negative | negative | yes |
| a6130de4-5db9-4640-b832-f3773693868a | Montpellier |  |  |  |  |  |  |  |  |  |  | 14.08225 | 17 | 1230.591195 | 1271 |  |  |  |  | 1244.673445 | 1288 | negative | negative | yes |
| 7c767017-c67b-48e4-9360-b7a26631ce21 | Montpellier |  |  |  |  |  |  |  |  |  |  | 35.547968 | 40 | 1371.973085 | 1416 |  |  |  |  | 1407.521053 | 1456 | negative | negative | yes |
| f38c7473-a8d0-4858-9508-06aba9456e32 | Montpellier |  |  |  |  |  |  |  |  |  |  | 41.98945 | 49 | 1473.488751 | 1531 |  |  |  |  | 1515.478201 | 1580 | negative | negative | yes |
| a1e87716-a3bb-4910-b539-4f076474c1be | Montpellier |  |  |  |  |  |  |  |  |  |  | 27.955256 | 32 | 1133.057107 | 1184 |  |  | 13.747407 | 15 | 1174.75977 | 1231 | negative | negative | yes |
| 1e8d8983-3474-470a-ba18-99a4c1a60533 | Montpellier |  |  |  |  |  |  |  |  |  |  | 116.420775 | 135 | 1653.870398 | 1753 |  |  | 1.849443 | 2 | 1772.140616 | 1890 | negative | negative | yes |
| a472ca12-f778-421a-a2c4-8e79ca756128 | Montpellier |  |  |  |  |  |  |  |  |  |  | 248.488395 | 297 | 2863.623656 | 2979 |  |  | 3.854979 | 4 | 3115.96703 | 3280 | negative | negative | yes |
| b2d9fb45-4e06-4f6b-9a86-1e0dbe56b407 | Montpellier |  |  |  |  |  |  |  |  |  |  | 16.873084 | 23 | 1504.339048 | 1559 |  |  |  |  | 1521.212132 | 1582 | negative | negative | yes |
| 60e3630d-f83a-43dd-9cd0-d92593d48fc6 | Montpellier |  |  |  |  |  |  |  |  |  |  | 91.955031 | 105 | 2640.718916 | 2760 |  |  | 57.083362 | 61 | 2789.757309 | 2926 | negative | negative | yes |
| 7b3b7aee-8213-4f6c-9fd9-ae29adf988d0 | Montpellier |  |  |  |  |  |  |  |  |  |  | 87.031301 | 104 | 1252.426958 | 1297 |  |  | 1.930548 | 2 | 1341.388807 | 1403 | negative | negative | yes |
| d9618bbd-2ffd-4e0b-ab70-1c2107465493 | Montpellier |  |  |  |  |  |  |  |  |  |  | 319.168522 | 355 | 1389.990051 | 1431 |  |  | 2.875685 | 3 | 1712.034258 | 1789 | negative | negative | yes |
| 625af97e-deeb-493c-9639-cc8a762bd04d | Montpellier |  |  |  |  |  |  |  |  |  |  | 103.242782 | 114 | 1565.134674 | 1613 |  |  | 0.953536 | 1 | 1669.330992 | 1728 | negative | negative | yes |
| 04bdb398-e300-48d1-8e50-43f6a80b7a95 | Montpellier |  |  |  |  | 0.599938 | 1 |  |  |  |  | 35.415622 | 41 | 1670.331444 | 1718 |  |  | 0.896956 | 1 | 1707.24396 | 1761 | negative | P. malariae | no |
| ed260be2-a702-4025-97a4-8863b9517927 | Montpellier |  |  |  |  |  |  |  |  |  |  | 68.841581 | 79 | 2345.77956 | 2424 |  |  | 8.719217 | 10 | 2423.340358 | 2513 | negative | negative | yes |
| 0bfe89a9-0f26-4437-91ab-52ad11d7a512 | Montpellier |  |  |  |  |  |  |  |  |  |  | 33.67527 | 39 | 1719.43446 | 1775 |  |  |  |  | 1753.10973 | 1814 | negative | negative | yes |
| f49e4e27-0334-471a-a721-cd5b50c36377 | Montpellier |  |  |  |  |  |  |  |  |  |  | 61.149128 | 72 | 2503.002186 | 2586 |  |  |  |  | 2564.151314 | 2658 | negative | negative | yes |
| c227d958-9a2b-4db9-87cc-80d24a59f655 | Montpellier |  |  |  |  | 0.839084 | 1 |  |  |  |  | 72.734773 | 88 | 2003.522255 | 2101 |  |  |  |  | 2077.096112 | 2190 | negative | P. malariae | no |
| 797bca5b-bd11-40a2-b050-295550e99e9b | Montpellier |  |  |  |  |  |  |  |  |  |  | 56.503135 | 65 | 2091.012262 | 2211 |  |  | 2.691368 | 3 | 2150.206765 | 2279 | negative | negative | yes |
| a9fd654a-51bf-4034-a9fc-8540bc91b1dd | Montpellier |  |  |  |  |  |  |  |  |  |  | 137.122661 | 152 | 2123.841701 | 2231 |  |  | 14.192597 | 15 | 2275.156959 | 2398 | negative | negative | yes |
| 5737978a-4557-4242-a33c-a16f73078caf | Montpellier |  |  |  |  |  |  |  |  |  |  | 9.710308 | 12 | 1989.297935 | 2058 |  |  | 0.952467 | 1 | 1999.96071 | 2071 | negative | negative | yes |
| 3c014942-e609-4edb-bcee-2b8af29edb40 | Montpellier |  |  |  |  |  |  | 0.718159 | 1 |  |  | 237.287908 | 259 | 3225.066982 | 3346 |  |  | 11.294805 | 12 | 3474.367854 | 3618 | negative | P. ovale | no |
| 51ec8019-e57f-4928-8485-1131cc3ecbb0 | Montpellier |  |  |  |  |  |  |  |  |  |  | 48.447305 | 54 | 1930.254149 | 2021 |  |  | 4.639512 | 6 | 1983.340966 | 2081 | negative | negative | yes |
| f4d731ad-1133-4d98-9c8f-a84655da7eff | Montpellier |  |  |  |  | 0.780245 | 1 |  |  |  |  | 155.663677 | 174 | 2192.39617499999 | 2290 |  |  | 9.453009 | 10 | 2358.29310599999 | 2475 | negative | P. malariae | no |
| f6c48215-3f4f-4926-94de-0eb9a5116df2 | Montpellier |  |  |  |  |  |  |  |  |  |  | 47.919592 | 54 | 1257.4818 | 1298 |  |  | 6.641013 | 7 | 1312.042405 | 1359 | negative | negative | yes |
| 95cded39-8e7b-4d0c-994c-06323636a1c3 | Montpellier |  |  |  |  | 0.681737 | 1 |  |  |  |  | 7.754812 | 10 | 860.064145 | 896 |  |  |  |  | 868.500694 | 907 | negative | P. malariae | no |
| 02aaf9a9-d37c-4520-9de6-c77f8dd23f08 | Montpellier |  |  |  |  |  |  |  |  |  |  | 32.599298 | 35 | 1172.794393 | 1212 |  |  |  |  | 1205.393691 | 1247 | negative | negative | yes |
| a84942de-e66e-43c9-8d90-b5dae02b5128 | Montpellier |  |  |  |  | 1.552594 | 2 |  |  |  |  | 12.819257 | 15 | 1582.292327 | 1634 |  |  | 3.22122 | 4 | 1599.885398 | 1655 | negative | P. malariae | no |
| b26047fa-c62b-4fde-bb4f-0ef81f96cea8 | Montpellier |  |  |  |  |  |  |  |  |  |  | 81.895586 | 91 | 1778.860468 | 1833 |  |  | 1.914276 | 2 | 1862.67033 | 1926 | negative | negative | yes |
| 4c300cee-a4e7-4c19-ad57-7ba49852f1dd | Montpellier |  |  |  |  | 5.536764 | 7 |  |  |  |  | 81.986722 | 92 | 1955.982676 | 2020 |  |  | 0.958407 | 1 | 2044.464569 | 2120 | negative | P. malariae | no |
| 2e7e36e0-f6c9-4b05-bba2-43fb568aed46 | Montpellier |  |  |  |  |  |  |  |  |  |  | 32.286614 | 35 | 1379.413888 | 1420 |  |  | 2.907737 | 3 | 1414.608239 | 1458 | negative | negative | yes |
| f386290a-6785-4c86-8d15-126a01485995 | Montpellier |  |  |  |  | 4.36804 | 5 |  |  |  |  | 139.116141 | 156 | 1715.027616 | 1808 |  |  | 8.517139 | 9 | 1867.028936 | 1978 | negative | P. malariae | no |
| 3edf13eb-38ea-4d9d-944c-5f3572baadd3 | Montpellier |  |  |  |  |  |  |  |  |  |  | 96.527315 | 106 | 1233.932235 | 1275 |  |  | 2.754892 | 3 | 1333.214442 | 1384 | negative | negative | yes |
| 1f8ef578-b841-4beb-9805-8900b542aa32 | Montpellier |  |  |  |  |  |  |  |  |  |  | 22.95295 | 25 | 1722.591616 | 1779 |  |  | 1.777604 | 2 | 1747.32217 | 1806 | negative | negative | yes |
| 08935d89-76aa-4ccd-ac4d-4c61970ec13d | Montpellier |  |  |  |  |  |  |  |  |  |  | 123.880164 | 143 | 1628.859385 | 1682 |  |  | 3.244289 | 4 | 1755.983838 | 1829 | negative | negative | yes |
| 6c0072f6-32a6-4b4a-876d-150cd082adbc | Montpellier |  |  |  |  | 9.271444 | 12 |  |  |  |  | 138.910964 | 155 | 1840.537916 | 1966 |  |  | 1.898959 | 2 | 1990.619283 | 2135 | negative | P. malariae | no |
| 5c9995d6-85ef-4b2a-a623-12ede73793e6 | Montpellier |  |  |  |  | 3.66365 | 5 |  |  |  |  | 126.222552 | 144 | 2077.252602 | 2177 |  |  | 3.386298 | 4 | 2210.525102 | 2330 | negative | P. malariae | no |
| 4699657c-1689-4fcd-a854-0dcd896e8e62 | Montpellier |  |  |  |  |  |  |  |  |  |  | 17.501431 | 22 | 1397.955984 | 1453 |  |  |  |  | 1415.457415 | 1475 | negative | negative | yes |
| b06c7ee6-3d45-4398-9a34-4cbc5e1b1b6a | Montpellier |  |  |  |  |  |  |  |  |  |  | 10.537562 | 12 | 1131.531519 | 1166 |  |  |  |  | 1142.069081 | 1178 | negative | negative | yes |
| 0993597b-d87b-4e6f-8529-65490f4fadad | Montpellier |  |  |  |  |  |  |  |  |  |  | 105.878196 | 115 | 1293.403851 | 1332 |  |  | 0.954428 | 1 | 1400.236475 | 1448 | negative | negative | yes |
| 8d936d20-7d03-4abe-a0f8-584969f8e7cc | Montpellier |  |  |  |  | 0.770363 | 1 |  |  |  |  | 38.733222 | 43 | 1209.844961 | 1247 |  |  |  |  | 1249.348546 | 1291 | negative | P. malariae | no |
| d0095fc4-8dad-49c1-aab7-56f09b550f5d | Montpellier |  |  |  |  |  |  |  |  |  |  | 66.134401 | 75 | 991.708279999999 | 1025 |  |  | 1.826062 | 2 | 1059.668743 | 1102 | negative | negative | yes |
| b82c529c-05b7-410b-be5d-004223ff8573 | Montpellier |  |  |  |  |  |  |  |  |  |  | 45.266048 | 51 | 1270.286464 | 1311 |  |  | 0.952668 | 1 | 1316.50518 | 1363 | negative | negative | yes |
| 76d88e1e-2178-4df0-94a8-bb32e2fd8123 | Montpellier |  |  | 0.873961 | 1 |  |  |  |  |  |  | 90.697187 | 104 | 2422.52029500001 | 2544 |  |  | 4.866614 | 6 | 2518.95805700001 | 2655 | negative | P. falciparum | no |
| baf87ad3-ff7a-47dc-9bf5-6e222e8f6aac | Montpellier |  |  |  |  |  |  |  |  |  |  | 187.983448 | 206 | 2314.396799 | 2387 |  |  | 5.878032 | 7 | 2508.258279 | 2600 | negative | negative | yes |
| ce654a43-03b8-45af-a5a6-eb80ea0cc20b | Montpellier |  |  |  |  |  |  |  |  |  |  | 43.899241 | 49 | 1294.109049 | 1354 |  |  |  |  | 1338.00829 | 1403 | negative | negative | yes |
| 4379bec2-6e4a-49c1-9c24-7912714173a6 | Montpellier |  |  |  |  |  |  |  |  |  |  | 93.800374 | 103 | 1421.971537 | 1467 |  |  | 1.487303 | 2 | 1517.259214 | 1572 | negative | negative | yes |
| bcb6aea3-008a-4aa0-ac1c-b2ccfc2685ca | Montpellier |  |  |  |  |  |  |  |  |  |  | 70.648596 | 79 | 1655.414948 | 1718 |  |  | 1.735457 | 2 | 1727.799001 | 1799 | negative | negative | yes |
| 2542c675-93d8-4cd5-acb0-7175dec9c220 | Montpellier |  |  | 0.66607 | 1 |  |  |  |  |  |  | 101.157698 | 112 | 1560.696596 | 1617 |  |  | 1.859745 | 2 | 1664.380109 | 1732 | negative | P. falciparum | no |
| 560dbda4-655f-4645-9994-a196c33318d9 | Montpellier |  |  |  |  |  |  |  |  |  |  | 91.242105 | 101 | 1854.615894 | 1933 |  |  | 2.870495 | 3 | 1948.728494 | 2037 | negative | negative | yes |
| de4ca722-7177-43c6-8b94-64ebeec055fa | Montpellier |  |  |  |  |  |  |  |  |  |  | 31.553724 | 36 | 1893.075945 | 1967 |  |  | 7.066313 | 8 | 1931.695982 | 2011 | negative | negative | yes |
| 1f07a853-2f3d-4584-9491-0c95f79eff04 | Montpellier |  |  | 1.254397 | 2 |  |  |  |  |  |  | 110.643869 | 121 | 1635.317153 | 1707 |  |  |  |  | 1747.215419 | 1830 | negative | P. falciparum | no |
| dd90b249-d9a7-4a90-98c6-96c81e590252 | Montpellier |  |  |  |  |  |  |  |  |  |  | 145.014605 | 163 | 1693.386008 | 1747 |  |  |  |  | 1838.400613 | 1910 | negative | negative | yes |
| df03886e-1add-4ada-857d-2f65c5a17735 | Lille |  |  | 55.840251 | 66 |  |  |  |  | 1.558176 | 2 | 13.941842 | 17 | 1584.810341 | 1687 |  |  | 0.952162 | 1 | 1657.102772 | 1773 | P. falciparum | P. falciparum | yes |
| c29eb78d-b5a9-4173-be15-5fd322945a5a | Lille |  |  | 22.970943 | 27 |  |  |  |  | 1.785646 | 3 | 30.601952 | 36 | 1327.836803 | 1401 |  |  |  |  | 1383.195344 | 1467 | P. falciparum | P. falciparum | yes |
| 9f01d763-d656-4a38-9d4e-693fd2fbcdf9 | Lille | 0.657551 | 1 | 38.69347 | 46 |  |  |  |  | 0.712357 | 1 | 8.297329 | 11 | 1722.020518 | 1901 |  |  |  |  | 1770.381225 | 1960 | P. falciparum | P. falciparum | yes |
| 38351ffa-176e-403b-8ad8-e8ac771ba22c | Lille |  |  | 36.462142 | 44 |  |  | 1.855454 | 3 | 1.149319 | 2 | 28.27166 | 31 | 2051.442911 | 2183 |  |  |  |  | 2119.181486 | 2263 | P. falciparum | P. falciparum | yes |
| bb153be5-f2b9-42b1-b57a-e36ffc92346b | Lille | 0.592867 | 1 | 33.893892 | 41 |  |  |  |  |  |  | 2.4701 | 3 | 1413.536728 | 1497 |  |  |  |  | 1450.493587 | 1542 | P. falciparum | P. falciparum | yes |
| 7e2af601-68b2-40b3-ada3-be252c9c6369 | Lille | 2.982713 | 5 | 57.064107 | 71 |  |  |  |  |  |  | 10.924002 | 12 | 1557.235871 | 1671 |  |  |  |  | 1628.206693 | 1759 | P. falciparum | P. falciparum | yes |
| db048793-6772-4dda-a088-174e50dbc0d7 | Lille | 16.839085 | 26 | 29.607087 | 38 |  |  |  |  |  |  | 34.029602 | 43 | 1947.04274700001 | 2113 |  |  |  |  | 2027.51852100001 | 2220 | P. falciparum | P. falciparum | yes |
| 777e3457-8d19-4393-91bc-2ff9949c8f6e | Lille | 1.947713 | 3 | 24.875833 | 30 |  |  | 0.667132 | 1 |  |  | 8.113262 | 9 | 1299.807149 | 1367 |  |  | 2.487337 | 3 | 1337.898426 | 1413 | P. falciparum | P. falciparum | yes |
| 3823ed93-015d-4ce9-8255-9b123b7b5fd6 | Lille | 15.807956 | 22 | 14.969247 | 20 |  |  |  |  |  |  | 9.218021 | 13 | 1569.202901 | 1715 |  |  |  |  | 1609.198125 | 1770 | P. falciparum | B. divergens | no |
| 0157931b-0bf7-4b6e-b317-e4a649bde058 | Montpellier |  |  | 20.584615 | 23 |  |  |  |  |  |  | 31.864014 | 36 | 1203.452246 | 1243 |  |  | 0.729649 | 1 | 1256.630524 | 1303 | P. falciparum | P. falciparum | yes |
| bce2961e-cf15-4f4f-b6dd-67fe077ba68d | Montpellier |  |  | 49.940149 | 57 | 5.256958 | 7 |  |  |  |  | 21.252196 | 23 | 2514.855998 | 2605 |  |  | 3.853928 | 4 | 2595.159229 | 2696 | P. falciparum | P. falciparum | yes |
| 56a4727b-104c-45d6-8777-683c1a742b99 | Montpellier |  |  | 0.793384 | 1 | 0.747945 | 1 |  |  |  |  |  |  | 112.496949 | 120 |  |  |  |  | 114.038278 | 122 | P. falciparum | P. falciparum | yes |
| 38915a92-6a19-4492-95db-2229f0662851 | Montpellier |  |  | 3.355182 | 5 | 15.943319 | 20 |  |  |  |  | 24.314679 | 27 | 1610.273902 | 1668 |  |  | 1.675094 | 2 | 1655.562176 | 1722 | P. falciparum | P. malariae | no |
| 4a6e301c-70a0-4b19-9c28-52548bd20c47 | Montpellier |  |  | 22.614293 | 24 |  |  |  |  |  |  | 22.267777 | 26 | 1338.668449 | 1388 |  |  |  |  | 1383.550519 | 1438 | P. falciparum | P. falciparum | yes |
| fea611f3-0a47-4522-834b-6deb55f155de | Montpellier |  |  | 87.752136 | 95 |  |  |  |  |  |  | 33.415491 | 39 | 2571.073535 | 2682 |  |  | 0.960572 | 1 | 2693.201734 | 2817 | P. falciparum | P. falciparum | yes |
| 3822549c-0db7-4dfe-b13f-82015441cc45 | Montpellier |  |  | 152.205237 | 161 |  |  |  |  |  |  | 163.921848 | 188 | 4078.888013 | 4267 |  |  | 8.885117 | 10 | 4403.900215 | 4626 | P. falciparum | P. falciparum | yes |
| 350bf5dd-625c-4b38-891d-de51ead9fc94 | Montpellier |  |  | 106.226683 | 112 |  |  |  |  |  |  | 89.313663 | 104 | 2696.736259 | 2823 |  |  | 4.652162 | 5 | 2896.928767 | 3044 | P. falciparum | P. falciparum | yes |
| 8559dc03-83ad-4ff0-baca-60531dafe5ae | Montpellier |  |  | 107.659836 | 115 |  |  |  |  |  |  | 85.532422 | 97 | 2737.247386 | 2857 |  |  | 1.900114 | 2 | 2932.339758 | 3071 | P. falciparum | P. falciparum | yes |
| aa17824d-a8b9-43f2-aa6e-f24d28311929 | Montpellier |  |  | 67.362764 | 73 |  |  |  |  |  |  | 1.80644 | 2 | 462.72487 | 494 |  |  | 0.950802 | 1 | 532.844876 | 570 | P. falciparum | P. falciparum | yes |
| a410a2ef-2e60-4179-b617-d10bc58f0802 | Rouen |  |  | 15.530576 | 17 |  |  |  |  | 0.632641 | 1 | 16.060162 | 18 | 1381.338243 | 1432 |  |  | 0.942363 | 1 | 1414.503985 | 1469 | P. falciparum | P. falciparum | yes |
| 3337239e-1f13-4822-9fb5-174c93c9e5f9 | Rouen |  |  | 12.278051 | 15 | 1.309382 | 2 |  |  |  |  | 31.991062 | 36 | 1637.894713 | 1711 |  |  | 1.852322 | 2 | 1685.32553 | 1766 | P. falciparum | P. falciparum | yes |
| ca05ce64-ab38-40da-a091-1d0502764372 | Rouen |  |  | 93.321053 | 111 | 2.979961 | 4 |  |  |  |  | 17.457297 | 19 | 2460.279506 | 2572 |  |  | 0.938354 | 1 | 2574.976171 | 2707 | P. falciparum | P. falciparum | yes |
| ee0dff84-5ad7-47bc-84b2-d079aa2b960c | Rouen |  |  | 11.346228 | 18 | 7.714308 | 11 |  |  |  |  | 97.79908 | 120 | 2006.804512 | 2149 | 2.476247 | 4 | 5.500494 | 7 | 2131.640869 | 2309 | P. falciparum | P. falciparum | yes |
| aa5934a3-7b19-41cc-aea6-f49cde4c49b5 | Rouen |  |  | 12.152984 | 16 |  |  |  |  |  |  | 74.406515 | 84 | 1066.037265 | 1122 | 2.408899 | 4 | 4.597321 | 5 | 1159.602984 | 1231 | P. falciparum | P. falciparum | yes |
| e845ce2b-22af-4e17-b011-ab33fa266a83 | Rouen |  |  | 48.990087 | 53 |  |  |  |  |  |  | 17.265169 | 20 | 1462.978402 | 1549 |  |  |  |  | 1529.233658 | 1622 | P. falciparum | P. falciparum | yes |
| 98fa757e-8789-46d9-8e61-cdd5860418fd | Rouen |  |  | 20.003062 | 23 | 0.674564 | 1 |  |  |  |  | 11.360262 | 13 | 1276.325237 | 1338 |  |  | 0.811021 | 1 | 1309.174146 | 1376 | P. falciparum | P. falciparum | yes |
| 01c2f934-1ef9-437e-beda-b7280af782d4 | Rouen |  |  | 116.921941 | 124 |  |  |  |  |  |  | 10.165976 | 11 | 1164.242497 | 1213 |  |  | 1.50286 | 2 | 1292.833274 | 1350 | P. falciparum | P. falciparum | yes |
| a52735e4-66e1-4bce-b353-000768f93cb6 | Rouen |  |  | 333.070959 | 369 |  |  |  |  |  |  | 29.454882 | 34 | 2860.478418 | 2979 |  |  | 4.61423 | 5 | 3227.618489 | 3387 | P. falciparum | P. falciparum | yes |
| f5b4afdf-fd71-4ecc-867e-c3e88bf94dfc | Rouen | 15.165293 | 21 | 0.599605 | 1 |  |  |  |  |  |  | 5.161503 | 6 | 676.732007999999 | 852 |  |  |  |  | 697.658408999999 | 880 | P. falciparum | B. divergens | no |
| d1a2dc7b-fc7e-4a6b-86a6-ab61614ef3c5 | Rouen |  |  | 7.982464 | 13 | 1.783452 | 3 |  |  | 0.72524 | 1 | 2.754451 | 4 | 1085.649049 | 1165 |  |  |  |  | 1098.894656 | 1186 | P. falciparum | P. falciparum | yes |
| ba2dfdc5-d79b-43a0-9762-ee2fe8b83295 | Rouen |  |  | 83.875742 | 104 | 0.748748 | 1 |  |  |  |  | 1.104135 | 2 | 1079.956983 | 1128 |  |  |  |  | 1165.685608 | 1235 | P. falciparum | P. falciparum | yes |
| 654fe05d-f1b4-4b33-9b5a-eb9cd8ff9471 | Toulouse |  |  | 14.227999 | 18 |  |  |  |  | 44.499336 | 57 | 47.465812 | 56 | 1497.893952 | 1576 |  |  |  |  | 1604.087099 | 1707 | P. falciparum | P. vivax | no |
| 8de02896-7f00-4548-9f8b-7aa12af2163a | Toulouse |  |  | 40.03486 | 42 |  |  |  |  |  |  | 17.500488 | 20 | 1520.678655 | 1577 |  |  |  |  | 1578.214003 | 1639 | P. falciparum | P. falciparum | yes |
| fe454423-77d7-4087-969b-ef35d28c8cba | Toulouse |  |  | 39.336101 | 43 |  |  |  |  |  |  | 9.081625 | 10 | 1558.946467 | 1622 |  |  |  |  | 1607.364193 | 1675 | P. falciparum | P. falciparum | yes |
| bc0529a8-0a8d-4d88-acd3-c38f28866eed | Toulouse | 3.083833 | 4 | 29.439592 | 32 |  |  |  |  |  |  | 20.398712 | 22 | 1341.582631 | 1395 |  |  |  |  | 1394.504768 | 1453 | P. falciparum | P. falciparum | yes |
| b1da3dd6-6c21-4899-a25e-19c24d1520b0 | Toulouse | 4.871893 | 6 | 36.61734 | 40 |  |  |  |  |  |  | 12.741114 | 14 | 1197.146575 | 1252 |  |  |  |  | 1251.376922 | 1312 | P. falciparum | P. falciparum | yes |
| b5ac18b2-3409-4bee-a5c8-0b3fbe521f5c | Toulouse |  |  | 28.587929 | 30 |  |  |  |  |  |  | 20.124067 | 22 | 1397.411506 | 1455 |  |  |  |  | 1446.123502 | 1507 | P. falciparum | P. falciparum | yes |
| 742f72f9-d8ad-4b12-92ba-0853ca3d2424 | Saint-Louis |  |  | 8.698355 | 11 |  |  | 2.166863 | 3 | 8.600192 | 11 | 5.939656 | 7 | 556.493075 | 585 |  |  |  |  | 581.898141 | 617 | P. falciparum | P. falciparum | yes |
| f31e62e6-3961-44b4-9670-b47b131e29e4 | Saint-Louis |  |  | 0.569466 | 1 |  |  |  |  |  |  | 7.656621 | 10 | 558.818993999999 | 597 |  |  | 0.934464 | 1 | 567.979544999999 | 609 | P. falciparum | P. falciparum | yes |
| 5089bb59-d789-413a-bb48-5eb569071185 | Saint-Louis |  |  | 3.395072 | 5 | 1.402307 | 2 |  |  | 0.865189 | 1 | 7.884112 | 10 | 573.409686999999 | 603 |  |  | 0.696634 | 1 | 587.653000999999 | 622 | P. falciparum | P. falciparum | yes |
| 8ce243bd-1669-46af-aed0-61b9231fd5de | Saint-Louis |  |  | 20.144948 | 24 |  |  |  |  |  |  | 0.775899 | 1 | 522.931871 | 553 |  |  | 0.951034 | 1 | 544.803752 | 579 | P. falciparum | P. falciparum | yes |
| e47d73f4-ad3a-4e9c-9749-9e57440aa018 | Saint-Louis |  |  | 16.17657 | 19 |  |  |  |  |  |  | 11.812508 | 13 | 565.374514 | 598 |  |  | 0.911945 | 1 | 594.275537 | 631 | P. falciparum | P. falciparum | yes |
| d92e581c-c168-4cc2-81f8-7cdf9cb88669 | Lille |  |  | 15.271678 | 23 |  |  | 3.099 | 5 | 1.417667 | 2 | 19.989341 | 24 | 1530.435171 | 1629 |  |  |  |  | 1570.212857 | 1683 | P. malariae | P. falciparum | no |
| 45a28240-9585-4518-9a2a-de30617526f0 | Rouen | 1.543716 | 2 | 3.53537 | 6 | 7.78744 | 11 |  |  |  |  | 63.962928 | 75 | 1542.272586 | 1663 |  |  | 5.247851 | 7 | 1624.349891 | 1764 | P. malariae | P. malariae | yes |
| f1f3baeb-54fc-4c22-931d-c7ef85b44cf0 | Rouen |  |  | 13.073307 | 18 | 20.019093 | 26 | 2.089681 | 3 |  |  | 45.532958 | 52 | 3259.096264 | 3423 |  |  | 1.923407 | 2 | 3341.73471 | 3524 | P. malariae | P. malariae | yes |
| cf8a3025-c5b8-43e9-8a66-0367abdaa840 | Rouen | 0.783371 | 1 | 22.687896 | 28 | 0.741956 | 1 |  |  |  |  | 21.122398 | 25 | 2072.316993 | 2168 |  |  |  |  | 2117.652614 | 2223 | P. malariae | P. falciparum | no |
| 12fb383a-25a4-4d22-8d2c-bb53e9be1dbe | Rouen |  |  |  |  | 17.929619 | 23 |  |  |  |  | 41.810989 | 48 | 1632.967546 | 1726 |  |  |  |  | 1692.708154 | 1797 | P. malariae | P. malariae | yes |
| a3ad4d5f-50f3-4c86-b6fc-99ec6a0b279a | Rouen |  |  | 0.618224 | 1 | 5.497567 | 8 |  |  |  |  | 35.499413 | 44 | 1444.546183 | 1528 |  |  | 5.151967 | 7 | 1491.313354 | 1588 | P. malariae | P. malariae | yes |
| 17c1bf4a-fbd8-4701-a7db-200260d16b50 | Rouen | 1.247543 | 2 |  |  | 4.254757 | 7 | 2.456751 | 4 | 0.572253 | 1 | 50.695658 | 59 | 1269.958142 | 1519 |  |  | 5.212452 | 6 | 1334.397556 | 1598 | P. malariae | P. malariae | yes |
| f135afae-4250-479e-bb25-4a10db596b2e | Rouen | 0.837094 | 1 | 0.716059 | 1 | 12.19935 | 17 |  |  | 1.31007 | 2 | 26.55186 | 32 | 1588.81795 | 1755 |  |  |  |  | 1630.432383 | 1808 | P. malariae | P. malariae | yes |
| ab60ceb4-50ff-4cad-ad09-749b43259879 | Rouen |  |  | 26.65344 | 33 | 5.122681 | 6 |  |  |  |  | 24.513228 | 29 | 2451.473488 | 2544 |  |  | 1.888614 | 2 | 2509.651451 | 2614 | P. malariae | P. falciparum | no |
| 150323cc-c722-41bd-9fbf-584f43fc9f24 | Rouen |  |  |  |  | 3.015956 | 4 |  |  | 1.451771 | 2 | 15.822916 | 17 | 372.369636 | 388 |  |  |  |  | 392.660279 | 411 | P. malariae | P. malariae | yes |
| fea0ca03-af6d-47e0-af91-729df4dc5c1f | Rouen |  |  | 0.705162 | 1 | 16.688993 | 20 |  |  |  |  | 33.310097 | 38 | 1654.379092 | 1758 |  |  | 1.864823 | 2 | 1706.948167 | 1819 | P. malariae | P. malariae | yes |
| ceb3bd55-8aa1-4107-a640-237950940cc4 | Rouen |  |  | 0.65652 | 1 | 5.799712 | 8 |  |  |  |  | 10.386673 | 12 | 437.025567 | 455 |  |  |  |  | 453.868472 | 476 | P. malariae | P. malariae | yes |
| c996016f-2874-43a9-b2d6-185932fed691 | Toulouse |  |  | 2.492576 | 3 | 18.653165 | 21 |  |  |  |  | 39.074804 | 42 | 1006.371097 | 1054 |  |  |  |  | 1066.591642 | 1120 | P. malariae | P. malariae | yes |
| 9fcf43d8-df0a-4ebc-a340-3d9802f1b75f | Toulouse |  |  | 1.462236 | 2 | 16.422851 | 19 |  |  |  |  | 81.161373 | 89 | 945.711333 | 981 |  |  | 1.8515 | 2 | 1046.609293 | 1093 | P. malariae | P. malariae | yes |
| fbeb3364-20df-40f6-94a2-1af040abefb4 | Toulouse |  |  | 2.334521 | 3 | 23.663146 | 26 |  |  |  |  | 31.231378 | 34 | 1131.71617 | 1177 |  |  | 1.922516 | 2 | 1190.867731 | 1242 | P. malariae | P. malariae | yes |
| 8bc5492a-ee56-4220-8996-730e48be0425 | Toulouse |  |  | 14.494387 | 18 | 4.642135 | 6 |  |  |  |  | 75.572706 | 82 | 1342.50716 | 1397 |  |  | 1.910098 | 2 | 1439.126486 | 1505 | P. malariae | P. falciparum | no |
| 40987fd9-a899-4f9c-b648-9ad12ef00f12 | Toulouse |  |  |  |  | 23.024082 | 25 |  |  |  |  | 27.079078 | 29 | 1256.354865 | 1305 |  |  | 0.964229 | 1 | 1307.422254 | 1360 | P. malariae | P. malariae | yes |
| 20e63977-5683-465c-8015-85f7d42f0340 | Saint-Louis |  |  |  |  | 6.83862 | 8 |  |  |  |  | 14.638791 | 18 | 290.695546 | 307 |  |  |  |  | 312.172957 | 333 | P. malariae | P. malariae | yes |
| 7861dda0-d9cd-466a-a435-6ce204d1a0f6 | Saint-Louis |  |  |  |  | 4.060937 | 5 |  |  |  |  |  |  | 121.648998 | 129 |  |  | 0.939977 | 1 | 126.649912 | 135 | P. malariae | P. malariae | yes |
| 8742bc07-53dc-4c58-9927-b5c62d0450ac | Saint-Louis |  |  |  |  | 10.895835 | 14 |  |  |  |  | 5.084038 | 7 | 349.295107 | 373 |  |  | 0.917801 | 1 | 366.192781 | 395 | P. malariae | P. malariae | yes |
| 22573196-b3ea-4898-ad50-d3a6ae354710 | Saint-Louis |  |  |  |  | 7.929097 | 10 |  |  |  |  | 1.839442 | 2 | 191.652116 | 205 |  |  | 0.958197 | 1 | 202.378852 | 218 | P. malariae | P. malariae | yes |
| b4cef84d-1de6-4723-a91b-81c32fdc6d69 | Saint-Louis |  |  |  |  | 32.500783 | 39 |  |  |  |  | 17.828755 | 22 | 933.948349999999 | 1048 |  |  | 1.887024 | 2 | 986.164911999999 | 1111 | P. malariae | P. malariae | yes |
| ad8a806e-130d-43d6-ad4e-d4c1a331c7a2 | Lille | 5.084734 | 8 | 9.137376 | 13 |  |  | 16.282496 | 23 | 0.732396 | 1 | 13.769573 | 17 | 1855.691797 | 2027 |  |  |  |  | 1900.698372 | 2089 | P. ovale | P. ovale | yes |
| 8565e362-dcd2-4b73-94d6-07248e189a5d | Rouen |  |  |  |  | 3.695605 | 5 | 3.661701 | 6 | 6.194755 | 9 | 26.157513 | 29 | 1497.28643 | 1554 |  |  | 2.880835 | 3 | 1539.876839 | 1606 | P. ovale | P. vivax | no |
| 2545625b-e311-46d9-8d16-f3b7baa6df06 | Rouen |  |  |  |  |  |  | 21.454685 | 26 |  |  | 35.048326 | 40 | 1676.461907 | 1753 |  |  | 0.877406 | 1 | 1733.842324 | 1820 | P. ovale | P. ovale | yes |
| 3b843cb9-37b7-4b47-8779-09859b60787f | Rouen |  |  | 2.486877 | 4 | 3.804307 | 6 | 4.600529 | 7 |  |  | 13.816164 | 16 | 1211.730132 | 1267 |  |  | 3.56838 | 4 | 1240.006389 | 1304 | P. ovale | P. ovale | yes |
| 56eec80a-1a03-4dfe-82f2-f6d6802c6253 | Rouen |  |  |  |  |  |  | 4.363693 | 6 | 6.57557 | 8 | 16.01803 | 17 | 938.566167 | 973 |  |  |  |  | 965.52346 | 1004 | P. ovale | P. vivax | no |
| f89ff3fc-ac96-45bb-9e15-d9c9d574ab17 | Rouen | 0.706631 | 1 |  |  | 3.195817 | 5 | 8.854307 | 13 | 1.700781 | 3 | 12.169642 | 17 | 1637.651883 | 1752 |  |  |  |  | 1664.279061 | 1791 | P. ovale | P. ovale | yes |
| 00dd6ce1-ec73-4384-8c40-c546cf592b4a | Rouen |  |  | 2.885413 | 4 | 1.129611 | 2 |  |  | 11.104827 | 15 | 18.461691 | 20 | 1536.040842 | 1599 |  |  | 0.944176 | 1 | 1570.56656 | 1641 | P. ovale | P. vivax | no |
| 2b967a38-2745-44c9-9d1d-1ff5febd2c04 | Rouen |  |  |  |  |  |  | 5.628527 | 8 | 10.923829 | 14 | 31.155204 | 35 | 1810.327647 | 1892 |  |  |  |  | 1858.035207 | 1949 | P. ovale | P. vivax | no |
| e2a4db29-05fa-4b00-9c64-16fdbdd98e8d | Rouen |  |  | 0.58733 | 1 |  |  | 1.157949 | 2 | 15.648108 | 19 | 38.476115 | 42 | 1639.872757 | 1715 |  |  | 3.255155 | 4 | 1698.997414 | 1783 | P. ovale | P. vivax | no |
| d5d71283-9771-443c-bf9b-8fefee329da0 | Rouen |  |  | 0.519632 | 1 |  |  | 1.384435 | 2 | 14.641049 | 18 | 30.11251 | 33 | 1700.573371 | 1766 |  |  | 1.670147 | 2 | 1748.901144 | 1822 | P. ovale | P. vivax | no |
| 65284d98-52b3-4f47-a7ac-d4d33027032b | Rouen |  |  |  |  |  |  |  |  | 20.93297 | 23 | 29.476947 | 32 | 1749.236247 | 1807 |  |  | 0.959801 | 1 | 1800.605965 | 1863 | P. ovale | P. vivax | no |
| f9176816-b729-4a6a-a330-e4b150f2da62 | Rouen |  |  |  |  |  |  |  |  | 25.065636 | 27 | 7.720421 | 10 | 1986.128109 | 2061 |  |  |  |  | 2018.914166 | 2098 | P. ovale | P. vivax | no |
| 85d94521-c66e-455b-9609-0d145603e819 | Rouen |  |  | 1.427616 | 2 |  |  | 1.469133 | 2 | 10.958787 | 14 | 12.032855 | 14 | 1451.528406 | 1510 |  |  |  |  | 1477.416797 | 1542 | P. ovale | P. vivax | no |
| 261229c7-4f4f-4f88-b516-663147e3d52d | Toulouse | 1.67135 | 2 | 1.843854 | 3 |  |  | 17.368574 | 22 |  |  | 44.83905 | 49 | 1090.47818 | 1138 |  |  | 0.954521 | 1 | 1157.155529 | 1215 | P. ovale | P. ovale | yes |
| 1df922de-886c-4b53-b2d2-2ed14b46be32 | Toulouse |  |  | 10.373282 | 13 |  |  | 6.676767 | 8 | 2.73118 | 4 | 23.509677 | 26 | 1344.881855 | 1412 |  |  | 2.816288 | 3 | 1390.989049 | 1466 | P. ovale | P. falciparum | no |
| 6c68ea51-3189-4f9b-949f-9dd79800b078 | Toulouse |  |  |  |  |  |  | 19.378144 | 23 | 1.927367 | 3 | 30.419438 | 34 | 1209.891209 | 1266 |  |  |  |  | 1261.616158 | 1326 | P. ovale | P. ovale | yes |
| 2db41446-a8df-4ef6-8b91-b423b90c2f2d | Toulouse |  |  | 4.880638 | 7 |  |  | 5.41824 | 7 | 3.805759 | 6 | 35.468816 | 39 | 1372.954343 | 1427 |  |  | 1.929729 | 2 | 1424.457525 | 1488 | P. ovale | P. ovale | yes |
| 21591fdd-c50f-4538-a632-21156a459e3c | Toulouse |  |  |  |  |  |  |  |  | 24.837234 | 28 | 35.733818 | 40 | 1869.051849 | 1938 |  |  | 0.950558 | 1 | 1930.573459 | 2007 | P. ovale | P. vivax | no |
| cfa61704-a2b5-46d0-8f19-77f3b5dd9877 | Saint-Louis |  |  | 1.156478 | 2 |  |  | 8.40477 | 12 | 2.59954 | 4 | 7.406027 | 11 | 572.956059 | 626 |  |  | 0.921439 | 1 | 593.444313 | 656 | P. ovale | P. ovale | yes |
| 8e3ab647-d58c-4363-837a-cd8320b96f2e | Saint-Louis |  |  |  |  |  |  | 3.444711 | 5 | 1.093454 | 2 | 25.696028 | 31 | 375.067536 | 420 |  |  | 5.594945 | 7 | 410.896674 | 465 | P. ovale | P. ovale | yes |
| 1316a526-f4c7-480f-a7ad-7a0366eeb428 | Saint-Louis |  |  |  |  |  |  | 5.644509 | 9 | 2.168603 | 3 | 12.205991 | 15 | 480.896844 | 519 |  |  | 1.697592 | 2 | 502.613539 | 548 | P. ovale | P. ovale | yes |
| bc3d7cc1-8bae-4e4a-8f0c-743bdaffeafe | Saint-Louis |  |  | 6.707753 | 10 | 5.35816 | 8 |  |  | 0.619074 | 1 | 17.805204 | 23 | 563.885438 | 615 |  |  |  |  | 594.375629 | 657 | P. ovale | P. falciparum | no |
| 21df0d71-676f-46a7-a74d-5647cd672fcb | Saint-Louis |  |  | 1.874303 | 3 |  |  | 3.09492 | 4 | 5.850776 | 8 | 7.666188 | 11 | 513.998917 | 552 |  |  |  |  | 532.485104 | 578 | P. ovale | P. vivax | no |
| af382d1e-4ba1-41e6-8778-937a342e33c5 | Lille |  |  | 9.977978 | 14 |  |  | 2.084528 | 3 | 24.149474 | 35 | 26.262769 | 30 | 1887.623771 | 2010 |  |  | 0.948077 | 1 | 1951.046597 | 2093 | P. vivax | P. vivax | yes |
| c71d5a6f-b3ee-4754-abc1-48b92b3f9ba1 | Lille |  |  | 20.634382 | 29 |  |  | 4.041969 | 6 | 15.586704 | 23 | 21.820485 | 25 | 1430.665991 | 1496 |  |  | 0.529465 | 1 | 1493.278996 | 1580 | P. vivax | P. falciparum | no |
| 2fee2501-3f09-4d4c-99d5-5bef4a158ae8 | Montpellier |  |  | 15.17387 | 19 |  |  | 14.528214 | 18 | 6.81308 | 8 | 14.480461 | 16 | 2886.776078 | 3042 |  |  |  |  | 2937.771703 | 3103 | P. vivax | P. falciparum | no |
| b872cd70-9350-4298-af30-1bed13a2874d | Rouen |  |  | 13.050692 | 16 | 0.847405 | 1 | 4.084716 | 6 |  |  | 42.281196 | 47 | 1099.134413 | 1166 |  |  | 0.76599 | 1 | 1160.164412 | 1237 | P. vivax | P. falciparum | no |
| b52ec725-eddb-4c93-bf74-a0369cbddb11 | Rouen |  |  |  |  |  |  | 18.867461 | 26 | 14.533559 | 21 | 170.359306 | 194 | 2734.715212 | 2856 |  |  | 6.716708 | 9 | 2945.192246 | 3106 | P. vivax | P. ovale | no |
| 08cf602a-6455-492c-a35e-95a4b33f3cdb | Rouen | 0.611653 | 1 | 6.2286 | 9 | 1.203354 | 2 | 2.663962 | 4 | 2.040556 | 3 | 41.505765 | 46 | 1670.42791 | 1776 |  |  | 3.776021 | 4 | 1728.457821 | 1845 | P. vivax | P. falciparum | no |
| b939ab89-9e8d-42c9-b1dc-289d1a7295aa | Rouen | 0.594482 | 1 | 5.736124 | 8 | 1.139554 | 2 | 2.422182 | 4 | 0.784217 | 1 | 51.521498 | 57 | 1561.178661 | 1631 |  |  |  |  | 1623.376718 | 1704 | P. vivax | P. falciparum | no |
| 8534b0e1-e010-4dc8-b813-49b6ccad9dc9 | Rouen | 1.932543 | 3 | 2.648742 | 4 | 1.801017 | 3 | 0.523058 | 1 | 6.343976 | 10 | 44.419307 | 50 | 1820.273752 | 1933 |  |  | 4.586612 | 6 | 1882.529007 | 2010 | P. vivax | P. vivax | yes |
| 64b0bb2e-d320-40b5-a0db-46bb6c272e1c | Rouen | 4.723473 | 7 | 3.81244 | 6 |  |  | 1.35802 | 2 |  |  | 24.846003 | 29 | 1735.940073 | 1828 |  |  | 2.104572 | 3 | 1772.784581 | 1875 | P. vivax | B. divergens | no |
| cd0a2434-870f-43de-801d-f499512b601f | Rouen |  |  | 5.354142 | 7 |  |  | 21.137312 | 30 | 0.684232 | 1 | 2.821172 | 4 | 1805.43736 | 1894 |  |  |  |  | 1835.434218 | 1936 | P. vivax | P. ovale | no |
| bf2adb59-a796-44fb-b59a-543d68e7a02e | Rouen |  |  | 1.549917 | 2 |  |  | 19.609618 | 22 |  |  | 7.154391 | 9 | 1578.382669 | 1716 |  |  | 1.708117 | 2 | 1608.404712 | 1751 | P. vivax | P. ovale | no |
| a43fd07f-c241-4f35-9a63-b9958cf81367 | Rouen |  |  |  |  |  |  | 6.029503 | 9 | 0.750702 | 1 | 8.379016 | 10 | 646.035037999999 | 720 |  |  | 1.94662 | 3 | 663.140878999999 | 743 | P. vivax | P. ovale | no |
| 5cc8d2ad-60c7-455e-866e-aba575f004ac | Rouen |  |  |  |  |  |  | 16.525755 | 22 | 2.953625 | 4 | 33.118106 | 37 | 1750.665815 | 1842 |  |  | 1.713104 | 2 | 1804.976405 | 1907 | P. vivax | P. ovale | no |
| 8ecd33db-2b17-465f-ba0a-dc04375532e3 | Toulouse |  |  |  |  |  |  | 5.502052 | 8 | 13.44753 | 19 | 14.496834 | 17 | 1402.668117 | 1463 |  |  | 0.939464 | 1 | 1437.053997 | 1508 | P. vivax | P. vivax | yes |
| 3184f2a3-58b1-4c9f-9bbc-ec592b092730 | Toulouse |  |  |  |  |  |  | 14.680517 | 20 | 7.548028 | 10 | 13.312353 | 17 | 1473.043331 | 1525 |  |  |  |  | 1508.584229 | 1572 | P. vivax | P. ovale | no |
| c2d62e8a-4948-4920-b014-5bcaccdd0330 | Toulouse |  |  |  |  |  |  | 9.230686 | 12 | 9.040616 | 11 | 28.715352 | 31 | 1202.801379 | 1255 |  |  | 1.485982 | 2 | 1251.274015 | 1311 | P. vivax | P. ovale | no |
| aec38426-f1db-4513-b3b3-b5f0edc1ea56 | Toulouse |  |  | 0.588032 | 1 |  |  | 18.623965 | 22 | 2.456939 | 4 | 34.937014 | 40 | 1592.412318 | 1678 |  |  |  |  | 1649.018268 | 1745 | P. vivax | P. ovale | no |
| da7276b3-c806-4081-aa04-9df39846dfbf | Toulouse |  |  | 0.50404 | 1 |  |  | 10.776338 | 14 | 12.935112 | 18 | 3.622914 | 4 | 1288.252575 | 1339 |  |  |  |  | 1316.090979 | 1376 | P. vivax | P. vivax | yes |
| 9cef7840-dfec-48a8-b262-8d203fe64736 | Saint-Louis |  |  | 11.639325 | 14 |  |  | 0.596171 | 1 | 2.962297 | 4 |  |  | 704.176732000001 | 750 |  |  | 0.871285 | 1 | 720.245810000001 | 770 | P. vivax | P. falciparum | no |
| c3c8f693-d7bf-4c25-90b1-4d43b90b1dc5 | Saint-Louis |  |  | 4.018475 | 6 | 0.558468 | 1 |  |  | 1.13964 | 2 |  |  | 533.173909 | 587 |  |  | 1.542046 | 2 | 540.432538 | 598 | P. vivax | P. falciparum | no |
| b0f233dd-4e00-448d-aa44-6472a4572490 | Saint-Louis |  |  | 12.660487 | 16 |  |  | 0.657583 | 1 | 2.232217 | 3 | 1.348705 | 2 | 582.533566 | 624 |  |  | 0.927087 | 1 | 600.359645 | 647 | P. vivax | P. falciparum | no |
| c9303a12-6b51-4023-8e03-8314089f8f32 | Saint-Louis |  |  |  |  | 1.826833 | 3 | 0.549681 | 1 | 2.078927 | 3 | 2.33133 | 3 | 373.561627 | 400 |  |  | 1.768639 | 2 | 382.117037 | 412 | P. vivax | P. vivax | yes |
| ec3cc5af-a364-4d8c-a7c4-7d614edb55c1 | Saint-Louis |  |  |  |  |  |  | 0.796097 | 1 | 7.446068 | 11 | 7.945993 | 11 | 602.236425 | 659 |  |  | 3.687073 | 6 | 622.111656 | 688 | P. vivax | P. vivax | yes |
| e5f168be-ffcb-441d-9206-d775478c89b2 | Saint-Louis |  |  |  |  |  |  |  |  |  |  | 34.075666 | 42 | 648.651809000001 | 731 | 12.949341 | 16 | 1.715591 | 2 | 697.392407000001 | 791 | T. brucei | T. brucei | yes |
| 435252d2-45d4-4b6b-9331-330ae1e0dc77 | Lille |  |  |  |  |  |  |  |  |  |  | 78.488236 | 94 | 1539.667292 | 1655 | 5.294629 | 8 | 4.798954 | 6 | 1628.249111 | 1763 | T. brucei | T. brucei | yes |
| fe68c53b-a450-4f5b-af5e-9dc1ba1e83f5 | Toulouse |  |  |  |  |  |  |  |  |  |  | **109.45124** | **121** | **1016.450635** | **1058** | **17.071584** | **21** | **0.91295** | **1** | **1143.886409** | **1201** | T. brucei | T. brucei | yes |
| **Total** |  | 140.906567 | 197 | 2198.86378 | 2547 | 338.872709 | 430 | 326.630518 | 438 | 378.484112 | 499 | 7567.478166 | 8620 | 252507.340253 | 265095 | 40.2007 | 53 | 368.041212 | 417 | 263866.818017 | 278296 |  |  | 116 |

**Table S5:** Detailed results per smear on the test dataset of the RT-DETR algorithm
